# Translating Molecular Psychiatry: From Biomarkers to Personalized Therapies—A Narrative Review

**DOI:** 10.3390/ijms26094285

**Published:** 2025-05-01

**Authors:** Tudor-Florentin Capatina, Anamaria Oatu, Casandra Babasan, Simona Trifu

**Affiliations:** 1 Department of Psychiatry, “Titu Maiorescu” University, 031593 Bucharest, Romania; tudorcapatina98@gmail.com; 2 Department of Psychiatry, “Iuliu Hatieganu” University of Medicine and Pharmacy, 400012 Cluj-Napoca, Romania; oatuanamaria@yahoo.com (A.O.); casandrababasan@yahoo.com (C.B.); 3 Department of Neurosciences, “Carol Davila” University of Medicine and Pharmacy, 020021 Bucharest, Romania

**Keywords:** gene, neurotransmitter, genetic variations, CYP, schizophrenia, MDD, GWAS

## Abstract

In this review, we explore the biomarkers of different psychiatric disorders, such as major depressive disorder, generalized anxiety disorder, schizophrenia, and bipolar disorder. Moreover, we show the interplay between genetic and environmental factors. Novel techniques such as genome-wide association studies (GWASs) have identified numerous risk loci and single-nucleotide polymorphisms (SNPs) implicated in these conditions, contributing to a better understanding of their mechanisms. Moreover, the impact of genetic variations on drug metabolisms, particularly through cytochrome P450 (CYP450) enzymes, highlights the importance of pharmacogenomics in optimizing psychiatric treatment. This review also explores the role of neurotransmitter regulation, immune system interactions, and metabolic pathways in psychiatric disorders. As the technology advances, integrating genetic markers into clinical practice will be crucial in advancing precision psychiatry, improving diagnostic accuracy and therapeutic interventions for individual patients.

## 1. Introduction

With the emergence of data that can be gathered all over the world in large quantities and with complex measurements, we can now develop many new techniques to help us treat all patients. New emerging technologies, such as artificial intelligence or machine learning, are used to identify patterns of interaction among variables. Moreover, electronic medical records can be used to improve the flow of information and communication between clinicians [1].

There have been a lot of novel discoveries in fields such as psychiatry, which could help us to understand the basis of the diseases that affect so many people’s lives. In this review, we will show that some quantifiable markers can be seen in psychiatric patients.

Major depressive disorder (MDD) is a highly prevalent and disabling psychiatric illness that affects hundreds of millions of individuals globally. Characterized by persistent sadness, loss of interest or pleasure, cognitive impairments, and somatic symptoms, MDD contributes substantially to the global burden of disease. It is associated with high rates of comorbidity, functional impairment, and suicide risk, making it a major public health concern. While environmental factors, such as early life stress, trauma, and chronic psychosocial adversity, are well-established contributors, genetic and biological components also play a significant role in individual susceptibility to depression.

Over the past decade, technological advances have enabled large-scale genome-wide association studies (GWASs), leading to the discovery of multiple genetic risk loci associated with MDD. These findings have deepened our understanding of the molecular mechanisms involved in depression and confirmed the polygenic nature of the disorder. No single gene is responsible for MDD; rather, the disorder arises from the cumulative effects of many common variants, each with a small effect size. These discoveries have prompted the development of polygenic risk scores (PRSs) to quantify an individual’s genetic predisposition to MDD.

The following sections of this study provide a structured exploration of the current landscape in MDD’s genetic elements and their implications, starting with an overview of the genetic risk loci identified for MDD through GWASs. It discusses the major loci implicated to date and their potential biological relevance, including associations with neuronal function, synaptic signaling, neurodevelopmental processes, and immune system regulation. Then, we introduce the concept of polygenic risk scores (PRSs), detailing the methodology behind their construction and their theoretical application in estimating genetic risk. We explain how PRSs are derived from GWAS summary statistics and used to aggregate small-effect variants across the genome. Despite offering new opportunities for understanding genetic vulnerability, current PRSs have limited clinical utility due to their low predictive power, reduced generalizability across populations, and exclusion of environmental and epigenetic influences. The following section critically examines these shortcomings, including concerns about sample diversity, phenotypic inconsistencies, and methodological challenges.

At the end of this study, we discuss future directions for research and potential clinical applications. We explore how combining PRSs with other biological, psychological, and environmental factors could lead to improved risk prediction and stratified prevention strategies. Importantly, this study also highlights the growing role of non-pharmacological treatments for MDD, such as repetitive transcranial magnetic stimulation (rTMS), and how genetic profiling may eventually help to personalize such interventions. Preliminary studies suggest that genetic markers (e.g., BDNF polymorphisms) may influence rTMS response, providing a foundation for future research on genetically informed treatment selection.

## 2. Genetic Markers

Psychiatric disorders strongly correlate with genetic factors. However, the genetic predisposition to comorbid psychiatric conditions does not follow a strictly Mendelian inheritance pattern [2]. Instead, there are a multitude of factors, including polygenic or oligogenic inheritance, incomplete penetrance, variable phenotypic expression, and genetic heterogeneity. Typically, predisposition arises from minor alterations in DNA that contribute to an individual’s vulnerability [3]. Genetic factors alone do not cause mental illness; rather, they interact with environmental influences [4]. Thus, the question is not which factor in particular is the cause of mental illness but how the interplay between genetic and environmental elements contributes to its development.

### 2.1. Major Depressive Disorder (MDD)

In MDD, we can see the influence of genes from the start. A large-scale genome-wide association study (GWAS) conducted on 1.2 million people found 178 genetic risk loci and 223 independently significant SNPs [5]. One of the most extensively studied genes involved in depression is SLC6A4, which is responsible for the reuptake of serotonin (5-HTT). Over time, it has attracted greater interest in treating depression and anxiety [6].

Genes involved in neurotransmitter systems such as dopaminergic (DAT, DRD, COMT), serotonergic (5-HTTLPR, HTR1A, HTR2A), and GABAergic (GABA, GAD, DBI) pathways have been implicated in conditions resembling anxiety. Moreover, it has been shown that variations in interleukin genes are associated with these mood disorders, suggesting a role for the immune system in these pathologies. However, even with these discoveries, the genetic bases of MDD and anxiety remain unclear. While anxiety appears to have a stronger genetic component than MDD, definitive evidence establishing a direct genetic link to these disorders is still lacking [7].

The age of onset is a variable that is often overlooked in discussions about MDD. Studies have shown that a significant locus (rs7647854 on chromosome 3) is associated with adult-onset MDD (age 27 years and older). Moreover, an earlier onset of MDD was linked to a greater genetic similarity with schizophrenia and bipolar disorder (BD), suggesting a potential link between early-onset depression and other psychiatric disorders [8].

**Figure 1:**
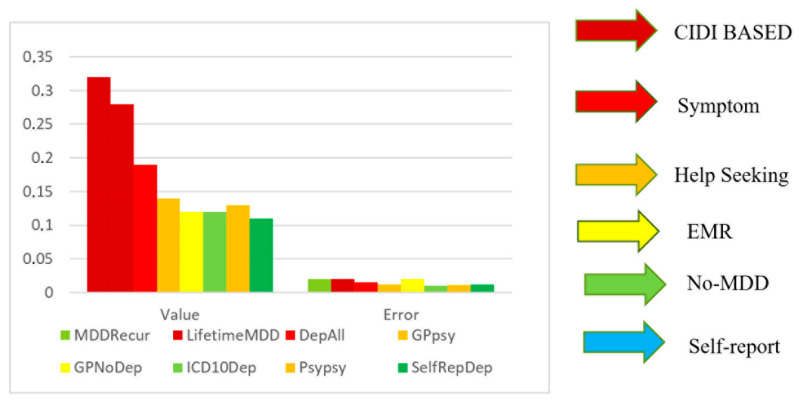
SNP-based heritability (h^2^SNP) estimates were obtained using the PCGC method for each definition of major depressive disorder (MDD) in the UK Biobank (methods). The heritability estimates, represented as h^2^ on the liability scale (h^2^(liab)), were adjusted to the liability scale using the observed prevalence of each depression definition in the UK Biobank as both the population and sample prevalence. Error bars indicate the standard errors of the estimates [9].

Research shows that a large portion of the genome contributes to shared genetic liability for other psychiatric disorders, indicating low genetic specificity for MDD. This trend remains consistent regardless of potential misestimations, regions of high linkage disequilibrium, or genomic complexity. This reinforces the idea that broader diagnostic criteria for MDD result in lower specificity. While polygenic risk scores have shown predictive value, this is primarily due to larger sample sizes rather than the identification of a distinct genetic signature for MDD. Even GWASs based on minimal phenotyping lack specificity for MDD, often identifying loci associated with general psychiatric susceptibility rather than MDD itself [9] (Figure 1).

### 2.2. Generalized Anxiety Disorder (GAD)

Twin studies show that the heritability range is between 30 and 50% [10]. When considering gender differences, women have significantly higher rates of all anxiety disorders, with rates being 1.5 to 2 times greater than those observed in men. The most obvious differences were seen in post-traumatic stress disorder (PTSD), GAD, and panic disorder (PD) [11].

**Figure 2:**
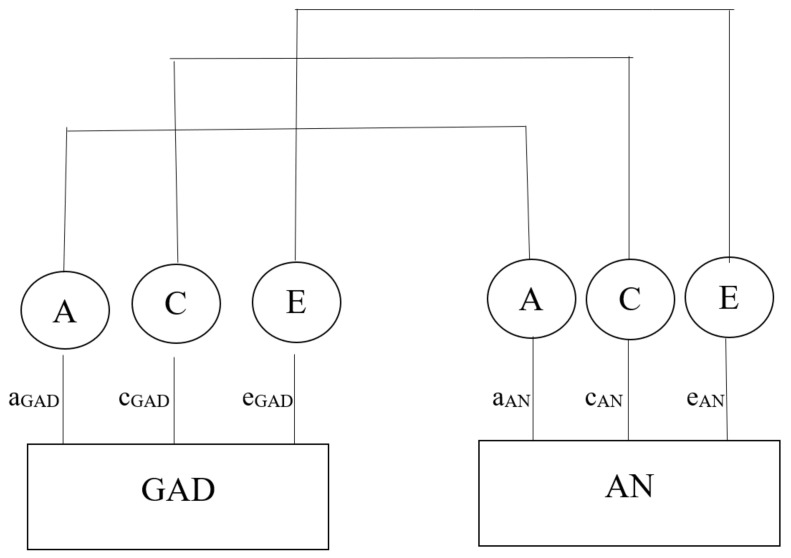
Bivariate structural equation model representing the genetic and environmental influences on generalized anxiety disorder (GAD) and anorexia nervosa (AN). The model includes A (heritability component), C (shared environmental component), and E (unique environmental component). Additionally, it depicts the inter-relationships between GAD and AN through the following correlation coefficients: r_a_ (genetic correlation), orc (shared environmental correlation), and r_e_ (unique environmental/error correlation) [14].

Interestingly, genetic predisposition appears to contribute to two distinct categories of anxiety disorders: one category includes panic disorder, GAD, and agarophobia, and the other is more specific to phobias [12]. Research suggests that depression and anxiety are influenced by different genetic factors in early childhood. However, as individuals age, genetic correlations between anxiety and depression become more pronounced [13]. Furthermore, anorexia nervosa has been found to have a modest genetic correlation with GAD, with a heritability value of 0.20. Alone, GAD has a heritability of 0.32, while anorexia nervosa has a heritability of 0.31. Despite this overlap, environmental factors play stronger roles in influencing each disorder individually than they do combined [14] (Figure 2).

Genetic similarities between GAD and neuroticism are the most significant factor linking the two conditions. The genetic correlation between them is approximately 80%, suggesting a strong shared genetic influence. This is not surprising, as they share substantial overlap in their underlying psychological and behavioral dimensions [15] (Table 1).

Furthermore, studies have shown that the frequency of the less active serotonin transporter (SLC6A4) polymorphic region (5-HTTLPR) is significantly higher in patients with both GAD and neuroticism than in healthy individuals [16,17].

The disruption of the G allele in the 5-hydorxytryptamine receptor 1A (5-HT1A), specifically the C-1019 polymorphism (rs6295), leads to reduced serotonergic signaling due to an enhanced negative feedback mechanism. This development has been linked to an increased prevalence of GAD in case-control studies [18].

Additionally, longer alleles of the monoamine oxidase A (MAOA) gene, characterized by an upstream variable number of tandem repeats (uVNTR) polymorphism, have been linked to a 12.6% greater likelihood of more severe GAD [19]. Moreover, a more active T allele of MAOA is significantly associated with GAD in females, but not in males, compared to control groups. This suggests the vital role of serotoninergic dysregulation in the pathophysiology of GAD [20].

A gene–environment interaction with 5-HTT haplotype has been identified, where exposure to childhood trauma activates genetic variants associated with increased transcriptional and enzymatic activity [21]. This interaction is linked to a heightened risk of anxiety sensitivity. In male populations exposed to early-life trauma, specific single-nucleotide polymorphisms (SNPs) have been shown to affect monoamine degradation, reducing its efficiency and, therefore, increase vulnerability to psychiatric disorders [22].

Research in mice has identified two loci: ofil-1 on chromosome 4 and ofil-2 on chromosome 7. These loci are associated with both anxiety and alcohol use behavior. Notably, ofil-1 showed a stronger linkage in female rats, suggesting potential sex-specific genetic influence on the comorbidity between anxiety and substance use [23].

### 2.3. Schizophrenia

The RNA-binding Hu protein family (ELAVL proteins) has been identified as a critical regulator of mRNA splicing in neuron-like cells, suggesting that it plays a key role in neuronal differentiation and function within the mammalian nervous system. These proteins are expressed at early stages of development in nearly all neurons, indicating their importance in neurodevelopmental processes [24]. It has already been shown that mutations in ELAVL4 are associated with an earlier onset of Parkinson’s disease [25], and the dysregulated expression of its known target, GAP43, has been observed in the frontal cortices and hippocampi of individuals with schizophrenia [26]. Moreover, decreased GAP-43 expression in the inner molecular layer and stratum radiatum of the CA2 has been found in the hippocampi of dead patients with schizophrenia and BD, suggesting that loss of neuroplasticity may be a cause of these psychiatric disorders [27,28].

Twin studies have demonstrated a concordance rate of approximately 50% for schizophrenia in monozygotic twins, underscoring the importance of both genetic predisposition and environmental factors in the etiology of the disorder [29].

Genes such as CSF2RA (colony-stimulating factor 2 receptor alpha) and IL3RA (interleukin-3 receptor alpha), which mediate proinflammatory responses via their respective ligands GM-CSF and IL-3, have been implicated in increasing susceptibility to perinatal infections. These inflammatory mechanisms are thought to contribute to the episodic nature of schizophrenia in adulthood [30,31]. Conversely, an increased prevalence of autoimmune disorders, such as Sjögren’s syndrome and celiac disease, has been reported prior to the onset of schizophrenia. This suggests that both inherited genetic factors and gene–environment interactions, including environmental exposures, may play a significant role in the development and progression of the disorder [32].

Deletions larger than 250 kb at the 2p16.3 locus, particularly those involving the 5′ end of the NRXN1 gene, have been identified as significant genetic alterations associated with schizophrenia. Additional evidence supports the role of deletions at the 3′ end of NRXN1 in contributing to the disorder, further implicating disruptions in NRXN1 in its pathogenesis [33]. The scientific literature also considers other genes associated with schizophrenia, as mentioned in Table 2 [34].

### 2.4. Bipolar Disorder (BD)

It is well established that psychiatric disorders have a significant genetic component, particularly in conditions such as schizophrenia and bipolar disorder (BD), where multiple genes have been implicated in disease onset. In BD, for instance, first-degree relatives have a 9% risk of also developing the disorder, which is nearly ten times higher than the risk in the general population. Additionally, children of parents with BD are approximately three times more likely to develop major depressive disorder (MDD) compared to those without a family history [35]. Monozygotic twins have a correspondence of 38–43% compared to 4.5–5.6% for dizygotic twins, further showing the importance of the genetic factor.

One promising area of investigation in the genetics of BD involves circadian rhythm genes. Sleep deprivation has been shown to temporarily alleviate depressive symptoms and stabilize mood in individuals with BD [36]. Moreover, many pharmacological treatments for BD influence circadian rhythms in both humans and animal models [37]. Key genes involved in this process include CLOCK and BMAL1, which form a heterodimer that drives a transcriptional feedback loop, regulating circadian rhythms over a 24 h cycle [38].

Another strong genetic candidate for BD is the single-nucleotide polymorphism (SNP) rs420259, located on chromosome 16p12, observed in both homozygous and heterozygous individuals. This SNP lies near several genes of potential significance: PALB2, involved in chromatin stabilization and nuclear structure maintenance; NDUFAB1, which encodes a component of the mitochondrial respiratory chain; and DCTN5, which interacts with DISC1, a gene already associated with psychiatric disorders. These findings suggest a multifactorial genetic influence on BD involving circadian regulation, mitochondrial function, and cellular structure maintenance [39].

The ANK3 gene, located on chromosome 10q21, encodes the ankyrin-G protein, a neuronal adaptor that plays a crucial role in regulating voltage-gated sodium channels, essential for proper neuronal signaling. Another key gene implicated in bipolar disorder (BD) is CACNA1C, situated on chromosome 12p13.33, which encodes the alpha-1C subunit of the L-type voltage-gated calcium channel. A specific variant of this gene, the rs1006737 single-nucleotide polymorphism (SNP), has been strongly associated with BD. Notably, ANK3 and CACNA1C have both been consistently identified across multiple studies as genetically correlated with BD, suggesting a shared role in its pathophysiology [40].

Recent genomic studies have further highlighted the overlap of genetic loci between BD and other major psychiatric disorders. Of the 64 loci identified in genome-wide analyses, 50 overlap with major depressive disorder (MDD), and 62 overlap with schizophrenia. These findings suggest that a substantial portion of the genetic variants implicated in BD are also involved in schizophrenia and MDD, suggesting the existence of a shared polygenic architecture across these disorders [41].

Genes influencing BD also seem to influence the response to treatment. For example, genes that are known to influence BPD include the following:

- ANK3 influences the structural constituent of the cytoskeleton and protein binding and bridging.
- CACNA1C influences enzyme binding and ion channel activity.
- SYNE1 is located on chromosome 6q25.2 and is seen in the cerebellar hemisphere and cerebellum, where it encodes multiple proteins with roles in synaptic plasticity and function.
- ODZ4(TENM4) is another gene identified in BPD that belongs to the tenascin family (teneurin subfamily), and it is located on chromosome 11q14.1, with a role in protein homodimerization activity.
- TRANK1(LBA1) is located on chromosome 3p22.2, and its expression is influenced by valproic acid. Moreover, its dysregulation can disrupt neuronal development and differentiation and the synaptic plasticity of other genes.

## 3. Pharmacogenomics

Personalized medicine customizes treatments, disease prevention, and health management to individual needs, with pharmacogenomics playing a pivotal role in enhancing outcomes and minimizing adverse effects [42].

Pharmacogenomics explores how genetic inheritance affects individual responses to drugs, integrating the fields of pharmacology and genomics. It holds promise for developing personalized medications tailored to a person’s unique genetic profile. While factors such as environment, diet, age, lifestyle, and overall health influence drug response, genetic makeup is considered the critical determinant for creating highly effective and safer treatments.

Drug responses, including both therapeutic and adverse effects, are complex traits influenced by multiple genes. Historically, the lack of understanding of all genes involved made it challenging to develop reliable genetic tests for predicting drug responses. However, the discovery of small genetic variations, specifically single-nucleotide polymorphisms (SNPs), revolutionized this field. These variations enable genetic testing to predict how an individual might react to specific medications.

Pharmacogenomics combines traditional pharmaceutical sciences, such as biochemistry, with genomic insights, including knowledge of genes, proteins, and SNPs. This integration has paved the way for personalized medicine, focusing on tailoring therapies to an individual’s genetic and biological characteristics [43].

### 3.1. CYP450 and Phenotypic Variations

Regarding drug metabolism, CYPs contribute to over 90% of documented enzymatic reactions and are predominantly expressed in the liver [44].

CYPs, among the most versatile catalysts in biochemistry, drive interindividual variations in drug responses. These variations arise from genetic and epigenetic differences, as well as environmental influences such as age, gender, nutrition, health conditions, and pathophysiological factors [45]. They can be inhibited or induced by co-administered drugs and circulating metabolites, impacting treatment outcomes through drug–gene interactions (DGIs), drug–drug interactions (DDIs), and drug–drug–gene interactions (DDGIs) [46]. Inhibitors of CYP enzymes include the antibiotic ciprofloxacin (CYP1A2 inhibitor) and the antidepressant fluvoxamine (potent CYP1A2 and CYP2C19 inhibitor, moderate or weak inhibitor for CYP3A4, CYP2C9, and CYP2D6) [47]. CYP enzyme inducers include carbamazepine (induces CYP3A and CYP1A2), phenytoin, phenobarbital, rifampin, and St. John’s wort (all CYP3A4 inducers) [48,49].

Other agents can affect CYP enzymes: caffeine and oral contraceptives are weak-to-moderate CYP1A2 inhibitors [50], while tobacco smoke, containing polycyclic aromatic hydrocarbons, weakly induces CYP1A2 by activating the aryl hydrocarbon receptor [51].

**Figure 3:**
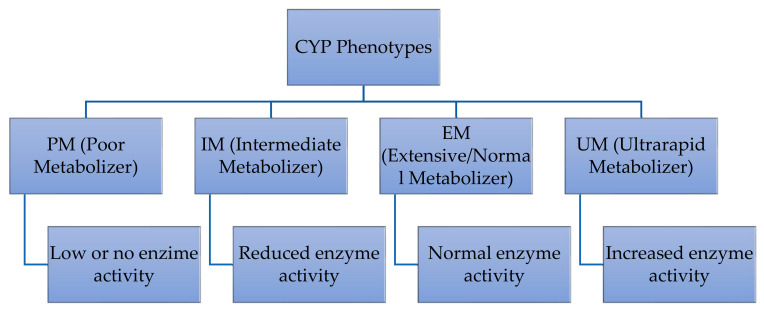
CYP metabolizer types.

Variations in CYP genes significantly influence individual differences in drug response and treatment outcomes. Genotyping and phenotyping tests for CYPs are becoming more common in clinical settings for identifying patients at risk of drug inefficacy or toxicity, enabling personalized treatment [52]. Four major CYP phenotypes arise from combinations of alleles with varying enzymatic activity levels: poor metabolizer (PM), intermediate metabolizer (IM), extensive (normal) metabolizer (EM), and ultrarapid metabolizer (UM) [53] (Figure 3). The ultrarapid metabolizer phenotype is linked to reduced therapeutic efficacy, while the poor metabolizer phenotype is associated with increased drug toxicity [54,55].

#### 3.1.1. Impact of CYP450 Genetic Variations on Antidepressant Response

Regarding antidepressant medication, genetic factors contribute to over 60% of the variability in drug responses and side effects associated with different classes of antidepressants, including selective serotonin reuptake inhibitors (SSRIs), serotonin–norepinephrine reuptake inhibitors (SNRIs), monoamine oxidase inhibitors (MAOIs), tetracyclic compounds, tricyclic antidepressants (TCAs), and noradrenergic–serotonergic modulators. Thus, when selecting the appropriate antidepressant and dosage, the patient’s genetic profile should be taken into consideration [56].

The CYP450 family comprises a broad group of enzymes involved in metabolizing varied drugs and xenobiotics, including antidepressants.

The CYP2D6 enzyme plays a role in metabolizing SSRIs (such as paroxetine, fluvoxamine, and fluoxetine), the SNRI venlafaxine, and the tricyclic antidepressant, amitriptyline [48]. Paroxetine and fluoxetine are strong inhibitors of CYP2D6, which can lead to an “iatrogenic poor metabolizer phenotype” or “phenocopy” when used alongside drugs such as venlafaxine, which are also metabolized by CYP2D6. Patients with this phenotype may be at an increased risk of toxicity due to higher plasma drug levels [57]. Individuals with reduced CYP2D6 function may have impaired metabolism of fluoxetine and paroxetine, leading to elevated blood levels of these drugs and an increased risk of adverse effects, such as developing suicidal ideation or antidepressant-induced mania, at the beginning of treatment; therefore, the close monitoring of all patients is required at the starting of antidepressant treatment [52,57].

Escitalopram, citalopram, and sertraline are primarily metabolized by CYP2C19. The CYP2C19*1 allele is linked to normal enzymatic function, while the CYP2C19*17 variant enhances CYP2C19 enzymatic activity. In contrast, the CYP2C19*2 and CYP2C19*3 variants result in loss of enzyme function [58,59].

Sim et al. [60] observed that individuals classified as poor CYP2C19 metabolizers showed fewer depressive symptoms compared to normal metabolizers. Additionally, recent research has identified a correlation between reduced CYP2C19 activity and the severity of depressive symptoms. These findings suggest that variations in CYP2C19 may influence susceptibility to depression [61,62].

The CYP1A2 enzyme metabolizes around 24% of antidepressant medications, such as escitalopram, venlafaxine, duloxetine, mirtazapine, and agomelatine [51]. Studies have shown that CYP1A2 polymorphisms influence antidepressant metabolism and treatment outcomes. Kuo et al. identified several SNPs, including rs2069521, rs4646425, and rs4646427, associated with altered escitalopram metabolism and increased adverse effects (e.g., fatigue, nausea) during early treatment stages [62]. Lin et al. linked CYP1A2 SNPs, such as rs4646425, rs2472304, and rs2470890, to slower responses to paroxetine treatment [63]. Additionally, the rs2470890 polymorphism has been suggested to influence remission rates during venlafaxine therapy.

#### 3.1.2. Impact of CYP450 Genetic Variations on Antipsychotic Response

A significant relationship has been observed between enzymes from the CYP family and the response to treatment with various medications, including antipsychotics [64].

Both aripiprazole and risperidone are metabolized primarily through CYP2D6 and CYP3A4. One study analyzed CYP2C19 and found a significant association between this enzyme and neurological side effects, including headaches, dizziness, drowsiness, and leg cramps, in healthy volunteers after a single dose of risperidone [65].

Most studies found no association between the CYP2D6 enzyme and the side effects of risperidone. However, some studies indicated that reduced CYP2D6 activity may influence risperidone-induced side effects. For instance, De Leon found that patients with the CYP2D6 poor metabolizer (PM) phenotype had a higher risk of moderate adverse reactions, including tremors, stiffness, hypersalivation, sedation, and sexual or urinary issues [66]. Two studies linked reduced CYP2D6 activity to elevated prolactin levels: in patients treated with risperidone, Schoretsanitis [67] observed this relationship in male patients, while Vandenberghe [68] reported it exclusively in women. Koller reported elevated prolactin levels in healthy volunteers with the CYP2D6 PM or IM phenotype following a single-dose treatment with aripiprazole [69]. Another study identified prolonged QTc intervals in patients with schizophrenia with CYP2D6 PM or IM phenotypes [70].

Clozapine, another atypical antipsychotic, is also metabolized by the cytochrome P450 enzymes in the liver, with CYP1A2 being the main enzyme involved in its metabolism [71]. CYP2D6, CYP3A4, and CYP2C19 are also involved in the metabolism of clozapine [72,73]. Increased CYP1A2 activity accelerates clozapine metabolism and shortens its half-life, while reduced CYP1A2 activity slows metabolism and extends its half-life [74]. The FDA recommends significantly reducing the clozapine dose when co-administered with strong CYP1A2 inhibitors due to decreased clearance. However, experts advise against co-prescribing inhibitors such as fluvoxamine or ciprofloxacin with clozapine due to the risk of fatal drug interactions [75,76].

Several SSRIs inhibit CYP2D6, impacting clozapine metabolism. Eggert, Crismon, and Dovjon reported no change in clozapine serum levels after initiating fluoxetine therapy [76]. In contrast, Joos et al. observed a clinically significant increase in clozapine levels 19 days after starting paroxetine therapy in a patient who was an extensive metabolizer (EM), highlighting their increased susceptibility to inhibitors [77]. A larger naturalistic study conducted by Centorrino et al. found that individuals treated with clozapine alone had significantly lower serum levels compared to those receiving clozapine with paroxetine, fluoxetine, or sertraline [78].

During inflammation, there is a release of cytokines that inhibit CYPs [79,80], including CYP1A2, increasing the risk of clozapine intoxication, particularly during infections such as pneumonia [81,82]. Reduced clozapine clearance during systemic inflammation, marked by fever or elevated C-reactive protein (CRP), leads to higher serum clozapine levels. Thus, elevated CRP levels can indicate that inflammation is responsible for increased clozapine concentrations [83].

Olanzapine is primarily metabolized by the CYP1A2 enzyme and, to a lesser extent, by the CYP2D6 enzyme. The *1F allele (rs762551) significantly affects olanzapine’s pharmacokinetics and efficacy by increasing the inducibility of CYP1A2. This heightens the risk of increased enzyme activity when exposed to factors that stimulate its expression [84,85]. Nicotine strongly induces CYP1A2, influencing olanzapine metabolism, especially in *1F allele carriers. Smokers with the *1F/*1F genotype may require increased olanzapine doses or close monitoring to ensure therapeutic effectiveness [86]. Markowitz and DeVane observed that concomitant treatment with the CYP1A2 inhibitor ciprofloxacin doubled serum olanzapine levels in a single patient, which returned to baseline upon discontinuing the antibiotic. The patient experienced no side effects from the elevated antipsychotic levels [87]. Callaghan and colleagues reported that simultaneous treatment with the CYP2D6 inhibitor fluoxetine caused statistically significant but clinically insignificant changes in olanzapine serum levels and clearance rates [88].

Quetiapine is primarily metabolized by the CYP3A4 enzyme. In a multiple-dose study, simultaneously administering the CYP3A4 inhibitor ketoconazole significantly increased serum quetiapine levels and its half-life [89]. A significant decrease in serum quetiapine concentration was observed, due to increased clearance, when it was administered together with phenytoin, a CYP inducer. Investigators attributed these pharmacokinetic changes to CYP3A4 induction, as quetiapine is predominantly metabolized by this enzyme [90].

#### 3.1.3. Impact of CYP450 Genetic Variations on Anxiolytic Response

Diazepam is primarily metabolized by the CYP2C19 enzyme. CYP2C9, CYP2B6, CYP3A4, and CYP3A5 are also involved in the metabolism of diazepam. A recent study conducted by Skryabin et al. demonstrated a meaningful association between the CYP2B6 phenotype and the pharmacokinetics of diazepam [91]. The findings of this study suggest that dose reduction may be needed for CYP2C19 and/or CYP2B6 poor metabolizers to prevent adverse reactions such as dependence on and tolerance to benzodiazepines. Carriers of the CYP2C19*2 allele, associated with reduced CYP2C19 activity, may have a higher risk of adverse effects due to slower metabolism and drug accumulation. Conversely, carriers of the CYP2C19*17 allele, linked to increased enzyme activity, may experience diminished diazepam efficacy at standard doses because of faster metabolism and lower plasma drug levels [92,93] (Table 3).

A recent study identified a link between CYP3A5 polymorphism and midazolam metabolism. Carriers of the CYP3A5 rs776746 T allele showed lower plasma midazolam concentrations compared to those with the C allele and may need higher doses to achieve sedation [94]. The CYP3A5*3 polymorphism has been linked to altered alprazolam metabolism. Park et al. demonstrated that homozygous CYP3A5*3 carriers may metabolize alprazolam more slowly, leading to higher plasma concentrations [95].

Regarding the CYP3A4 enzyme, individuals carrying the CYP3A4*22 allele might show decreased CYP3A4 enzyme activity and a slower rate of drug metabolism [96].

Furthermore, there are no currently recognized pharmacogenetic biomarkers for benzodiazepines such as bromazepam and lormetazepam [97].

### 3.2. Effects of CYP2D6 and CYP2C19 Variants on Neurotransmitter Regulation

CYP450 enzymes play a key role in maintaining cellular homeostasis by metabolizing endogenous compounds such as dopamine, serotonin, cortisol, testosterone, and progesterone [98,99]. Since dopamine and serotonin cannot cross the blood–brain barrier, CYP450 enzymes are present in the brain. Specifically, CYP2D6 in the brain catalyzes the conversion of tyramine into dopamine and 5-methoxytryptamine into serotonin [100]. These enzymes influence human behaviors, including personality traits and neuropsychiatric disorders such as schizophrenia, obsessive–compulsive disorder, and major depression, with CYP2D6 variations playing a significant role [61]. Extensive metabolizers are associated with lower anxiety and greater social success than poor metabolizers [101].

On the other hand, CYP2C19 is expressed in human fetal brain tissue but disappears after birth, indicating its role in brain neurodevelopment and its significant impact on adult depressive traits. Specifically, the absence of CYP2C19 is associated with a lower prevalence of depression [102,103].

A recent study investigated the genetic influence of CYP2D6 polymorphism on schizophrenia susceptibility, suggesting that CYP2D6 variations may affect the hippocampal white matter structure and dopamine neurotransmission, emphasizing the role of neuronal connectivity in schizophrenia pathophysiology [104].

The impact of gene variants on the metabolism of dopamine, serotonin, and, potentially, other neurotransmitters adds complexity to predicting how these variants affect a patient’s overall response to medications [105]. However, adhering to dosing recommendations tailored to an individual’s phenotype can help to optimize psychotherapy outcomes.

### 3.3. Challenges

Psycho-pharmacogenetic studies generally agree that genetic variations influence treatment outcomes for patients taking psychotropic medications, offering opportunities to optimize therapy. However, many findings in the field remain inconsistent. For example, while the Clinical Pharmacogenetics Implementation Consortium (CPIC) advises against using amitriptyline in CYP2D6 poor metabolizers [101,106], the FDA only issues a caution for the same patient group [57,107]. Thus, limited training and confidence among prescribers in interpreting pharmacogenomic data impede clinical application [108,109,110]. The inconsistent results of psychiatric genotyping studies, driven by small sample sizes, limited expertise, and differences in patient demographics, clinical factors, and environments, have confined the use of psychopharmacologic tests to specific disorders such as refractory schizophrenia [53].

In addition, the complexities of psychiatric disorders and inter-ethnic variations in drug response pose significant challenges to pharmacogenomic testing in psychiatric clinics [111].

To overcome the challenges mentioned, future efforts should aim to advance precision medicine based on personalized genetic profiles. Achieving this goal requires healthcare professionals to be well informed about genetic principles. Essentially, raising awareness of the role of allelic variants in clinical practice and implementing electronic genetic records could help to identify patients who would benefit from pharmacogenetic testing, leading to more tailored and effective psychiatric treatments [112].

## 4. Metabolists

### 4.1. Insulin Resistance and Psychiatric Impact

Insulin resistance has been increasingly recognized as a key factor not only in metabolic diseases but also in various psychiatric conditions, including depression and cognitive disorders. The complex relationship between insulin signaling and brain function suggests that disturbances in insulin regulation may contribute to the development and progression of mental health issues [113].

Depression and insulin resistance share a complex, bidirectional relationship, with each condition exacerbating the other. Both conditions are influenced by common mechanisms, such as inflammation, dysregulation of the HPA axis, and intestinal dysbiosis. Genetic evidence suggests that shared pathways exist between depression and insulin-related diseases, such as metabolic syndrome and type 2 diabetes, with insulin resistance potentially serving as a metabolic subtype of depression and offering a target for personalized treatments [114].

Insulin plays a crucial role in synaptic signaling and plasticity by regulating NMDA, AMPA, and GABA receptors, as well as long-term potentiation (LTP) and long-term depression (LTD), processes vital for learning and memory. These processes are modulated through the insulin/IGF-1 pathway, with insulin influencing synaptic strength and neural excitability. Insulin also regulates dendritic spine formation and synaptic development, playing a critical role in neuroplasticity [115,116,117]. Insulin’s influence on synapses is mediated through pathways such as the AKT–mTOR and RAC1–CDC42 pathways, with both involved in dendritic spine formation and the development of synaptic connections. This suggests that insulin plays an essential role in synaptic development and neuroplasticity, particularly regarding excitatory synapses [118].

Insulin also impacts glucose transport in the brain, regulating glucose uptake via transporters such as GLUT3 and GLUT4. During periods of increased neuronal activity, insulin facilitates GLUT4 translocation to meet the brain’s metabolic demands. In astrocytes, insulin binding triggers signaling pathways that regulate glucose metabolism, cell survival, and neuroprotection, emphasizing its crucial role in brain function [119,120,121,122].

However, insulin resistance may contribute to neuroprogression, accelerating cognitive decline and impairing brain function [123]. This is particularly evident in conditions such as bipolar disorder, where insulin resistance influences treatment resistance and exacerbates depressive symptoms [124]. Major depressive episodes in bipolar disorder are frequently accompanied by elevated levels of adrenocorticotropic hormone (ACTH), which stimulates cortisol secretion from the adrenal glands. This suggests that depressive symptoms may contribute to increased cortisol levels, promoting visceral fat deposition and worsening insulin resistance [125].

At the genetic level, disorders such as anorexia nervosa and schizophrenia have a negative genetic overlap with insulin-related diseases, while conditions such as ADHD and major depressive disorder have a positive overlap, suggesting that these disorders increase the genetic risk of developing metabolic conditions [126]. Additionally, diabetes is known to contribute to cerebrovascular disease, which can exacerbate dementia and reduce the effectiveness of cholinesterase inhibitors [127].

Second-generation antipsychotics, such as olanzapine and clozapine, are associated with an increased risk of prediabetes and T2D (type 2 diabetes), as they are commonly prescribed to treat schizophrenia and bipolar disorder [128,129]. Even patients with schizophrenia who have not yet received antipsychotic treatment, such as those in their first episode of psychosis, show a higher incidence of prediabetes and T2D, suggesting that the risk may not be solely due to medication. Studies have shown that individuals with schizophrenia may have an increased likelihood of having parents with T2D, indicating a potential genetic component to the comorbidity [130,131]. The connection between schizophrenia and T2D is multifactorial, influenced by genetic and environmental factors, as well as the use of antipsychotic drugs [132,133]. Postmortem studies of patients with schizophrenia show reduced expression of insulin receptors and signaling molecules (e.g., AKT, GSK3β, mTOR) in the frontal cortex, indicating impaired brain insulin sensitivity [134,135,136]. Research by Wijtenburg et al. found that patients with schizophrenia had poorer spatial memory performance and showed significant correlations between insulin resistance biomarkers, brain glucose levels, and memory performance [137].

In addition to lifestyle changes, pharmacological treatments may help to reduce the risk of developing antipsychotic-induced prediabetes and type 2 diabetes (T2D) in patients with schizophrenia [138]. A combination of the second-generation antipsychotic olanzapine with samidorphan, a μ-opioid antagonist, was approved by the FDA in June 2021. This combination has shown effectiveness in reducing medication-induced weight gain and metabolic dysfunction, which are linked to T2D [139]. In a 24-week phase 3 trial, the olanzapine–samidorphan combination significantly reduced weight gain and metabolic issues, with a 50% reduction in the incidence of metabolic syndrome compared to olanzapine alone in patients without metabolic syndrome [140]. Other treatments, such as the glucagon-like peptide-1 receptor agonist liraglutide, have shown promise in reducing the metabolic risks associated with antipsychotic medications. These emerging pharmacological strategies offer potential solutions for minimizing the metabolic risks linked to dopamine receptor-blocking antipsychotics in patients with schizophrenia [141].

Finally, intranasal insulin has emerged as a promising therapeutic approach for cognitive improvement, particularly in individuals with obesity or type 2 diabetes mellitus, offering potential benefits for addressing neurocognitive decline and improving memory [142,143,144].

### 4.2. Cholinesterases in Alzheimer’s Disease

The cholinergic system plays a central role in modulating both peripheral and central nervous system activity, primarily through the action of acetylcholine (ACh). This neurotransmitter is crucial for processes such as attention, memory, and sleep. At the core of this system is the enzyme type cholinesterase (ChE), particularly acetylcholinesterase (AChE), which regulates synaptic ACh levels by catalyzing its breakdown into choline and acetate. The enzyme has two key regions: the esteric subsite, involved in hydrolysis, and the anionic subsite (AS), which binds ACh and other cationic substances [145,146]. The esteric subsite features a catalytic triad (Ser200, Glu327, His440) responsible for ACh hydrolysis. [147] A cation–π interaction between ACh’s quaternary amine and an aromatic amino acid also aids the enzyme’s activity [146].

Cholinergic axons release acetylcholine (ACh), which then interacts with both metabotropic muscarinic receptors (M1) and ionotropic nicotinic receptors in the cerebral cortex. These receptors are involved in mediating various effects of ACh on cortical neurons [148,149]. ACh binding to postsynaptic M1 receptors leads to the suppression of potassium conductance, which helps to make the neuron more responsive to other excitatory inputs. This mechanism aligns with the modulatory role attributed to cholinergic input, which does not directly drive activity but instead primes the system to more efficiently process other stimuli [150]. Rodent models suggest that cholinergic activity is mostly based on volume transmission, where ACh diffuses over a wide area and affects neurons without forming direct synaptic connections. This view is supported by the observation that most cholinergic varicosities (or swellings along axons) in rodents do not form synapses [151,152].

The inhibition of acetylcholinesterase (AChE) is a cornerstone strategy for treating types of dementia such as Alzheimer’s disease (AD), where there is a progressive loss of acetylcholine (ACh) in the brain [153].

Acetylcholinesterase (AChE) inhibitors temporarily occupy the enzyme’s catalytic site (active site), where acetylcholine normally binds, without forming a covalent bond with the enzyme. The inhibition follows concentration-driven kinetics and is reversible and concentration-dependent. In contrast, carbamates, such as rivastigmine, are pseudo-irreversible inhibitors that form a covalent bond with the enzyme at the catalytic site. This bond eventually undergoes spontaneous hydrolysis, following pseudo-first-order kinetics [154].

The main challenge with AChE inhibitors that use these two mechanisms is their lack of CNS (central nervous system) selectivity, which is crucial for effective treatment. Acetylcholine is essential not only in the CNS but also in peripheral systems. When AChE inhibition reaches pharmacologically effective levels in the CNS, drugs without sufficient CNS selectivity can disrupt these critical peripheral functions, particularly in the gastrointestinal system. This was a major obstacle to using the high doses of conventional AChE inhibitors required to address severe acetylcholine deficits in the brain [155].

Jouvet is credited with conducting pioneering research suggesting that cholinergic signaling plays an essential role in generating REM (rapid eye movement) sleep. His work, followed by other researchers, laid the foundation for understanding the neurochemical pathways involved in sleep regulation [156]. In vivo pharmacological studies demonstrated that acetylcholine (ACh) receptor agonists, when injected into the brainstem, can induce REM sleep and muscle atonia (the paralysis that occurs during REM sleep, which prevents acting out dreams). This finding pointed to ACh’s critical role in initiating REM sleep [157]. Selective lesions of cholinergic cells in the brainstem resulted in reduced REM sleep duration, as well as decreases in both phasic (rapid eye movement) and tonic (muscle atonia) aspects of REM sleep. This suggests that pontine cholinergic neurons are pivotal for REM sleep generation. Selective lesions of cholinergic cells in the brainstem resulted in reduced REM sleep duration, as well as decreases in both phasic (rapid eye movement) and tonic (muscle atonia) aspects of REM sleep. This suggested that pontine cholinergic neurons are pivotal for REM sleep generation [158,159].

### 4.3. Microbiota Gut–Brain Axis

The gut microbiota is home to about 100 trillion microbes, which is significantly more than the number of human cells in the body (around 40 to 60 trillion cells) [160]. These microbes contain a gene set 150 times larger than the human genome, meaning that the microbial community contributes a massive amount of genetic diversity that can impact various physiological processes [161]. According to existing studies, there is strong evidence that depression is associated with a distinct gut microbiota composition. Shifts in specific microbial populations, particularly within the dominant phyla Bacteroidetes and Firmicutes, could potentially serve as biomarkers for depression or be targeted in microbiota-based therapies [162].

The gut microbiota have gained significant attention as a therapeutic target due to their potential for treating various conditions, including type II diabetes, autism, Alzheimer’s disease, and Parkinson’s disease This highlights the wide-ranging impact of the gut microbiome on overall health, not just for gastrointestinal issues but also for neurological and metabolic disorders [163,164].

Recent studies have linked gut microbiota imbalances to mental health disorders, particularly emphasizing depression. This has led to growing interest in exploring how modifying the gut microbiome might influence mental health [165]. High-throughput sequencing of fecal samples from depressed individuals has revealed that their microbial composition differs significantly from that of healthy individuals, suggesting that gut microbiota may play a role in the pathophysiology of depression [166]. Research comparing the brains of animals colonized with gut microbes (specific pathogen-free) and those that are germ-free has revealed significant downregulation of genes associated with microglia in the absence of gut microbiota. This suggests that the gut microbiota is essential for the proper maturation and function of microglia, which are crucial immune cells in the brain [167]. The structural components of bacteria in the gut interact with the immune system through Toll-like receptors (TLRs) present on various immune cells. These TLRs can be activated by gut microbes, influencing immune responses both locally in the gut and systemically [168]. In the central nervous system (CNS), cells such as astrocytes, microglia, and oligodendrocytes also express TLRs. These receptors play key roles in innate immunity, regulating CNS autoimmunity, neurodegeneration, and tissue damage, highlighting the significant connection between the gut microbiota and brain health [169,170].

The presence of oral bacteria such as *S. vestibularis* in the gut may contribute to neuroinflammatory processes or disruptions in brain signaling pathways, potentially offering insights into how the gut microbiome could influence psychiatric conditions such as schizophrenia [171]. The reduced activity of NMDA receptors and neurotrophic factor receptors could contribute to the cognitive and functional impairments often seen in schizophrenia, as these pathways are critical for synaptic plasticity, learning, and memory. The involvement of the gut microbiota in modulating these receptor pathways suggests that the microbiome may not only influence immune function and inflammation but also directly impact brain function and neurodevelopment in schizophrenia [172,173].

Fecal microbiota transplantation (FMT) is a technique for modifying the gut microbiota by transferring fecal material from a healthy individual to a patient. This method has been shown to cause or alleviate depression, providing evidence that modifying gut microbiota can influence mood and mental health [174,175]. While FMT has been explored in scientific studies on depression, it is infrequently utilized to treat psychiatric disorders. It was not until 2017 that Kang et al. used FMT to alleviate symptoms of autism, leading to continued improvements in both gastrointestinal and autism-related symptoms [176].

Prebiotics are non-digestible fibers that pass through the gastrointestinal tract and promote the growth of beneficial gut bacteria. Some common examples of prebiotics include galacto-oligosaccharides, fructo-oligosaccharides, inulins, and oligofructose. These compounds can influence the structure of the intestinal microbiota, promoting the growth of helpful bacteria such as Lactobacillus, Bacteroides, and Bifidobacterium. Importantly, the diversity and abundance of these bacteria are often reduced in individuals with depression [177,178]. A 2015 study by Andrew demonstrated that oligosaccharides found in human breast milk, such as 3′sialyllactose and 6′sialyllactose, may restrain anxiety development [179]. A 2019 meta-analysis by Richard et al. reviewed multiple clinical trials and found general support for the antidepressant and anti-anxiety effects of prebiotics. However, due to limitations in study design and sample sizes, further validation is needed to establish their efficacy definitively [180].

Lactobacillus rhamnosus has been shown to regulate excitation-induced plasma corticosterone levels, which are linked to stress responses and mood regulation. It may help to alleviate depression by modulating the secretion of hormones involved in emotional regulation, neuroplasticity, and stress responses. These effects are likely mediated through the vagus nerve, which connects the gut and brain, and the hippocampus, a key brain area involved in mood regulation and memory [181,182]. Bifidobacterium infantis also plays a role in depression relief, likely through influencing hormones such as acetylcholine and corticosterone. Similar to Lactobacillus rhamnosus, its effects may involve the vagal nerve, which acts as a conduit for communication between the gut and the brain, influencing emotional and stress responses [183,184].

There is anecdotal evidence suggesting that probiotics may improve both gut health and behavioral symptoms in individuals with ASD. Some mouse model studies have shown that probiotic treatments can reduce ASD-associated behaviors (such as social deficits and repetitive behaviors), and human trials have yielded mixed but generally promising results [185,186,187]. For instance, a 2020 review of studies on probiotic supplementation for individuals with ASD concluded that while probiotics show potential, we need more rigorous, standardized clinical trials to confirm their effectiveness. Key limitations in existing studies include small sample sizes and a lack of placebo controls [187].

### 4.4. Autophagy

Autophagy is a multifaceted process that entails recognizing, isolating, and delivering cellular cargo, such as damaged organelles and misfolded proteins, to lysosomes for degradation. Several mechanisms are involved in transporting this cargo to lysosomes, including de novo formation of autophagosomal vesicles that enclose the cargo and fuse with lysosomes (macroautophagy), the receptor-mediated transfer of cytosolic proteins directly across the lysosomal membrane (chaperone-mediated autophagy), and the invagination and scission of portions of the lysosomal membrane (microautophagy) [188]. Over 30 autophagy-related genes and their associated proteins play critical roles in these processes, including molecules involved in lipid conjugation, signal transduction, and forming elongating autophagosomes [189].

Remarkably, this unbiased analysis identified a substantial disruption in the expression of autophagy-related genes in the brains of patients with schizophrenia, especially in Brodmann area 22 (BA22) of the superior temporal cortex. This area has long been suspected to play a role in the development of schizophrenia [190,191]. Microarray analysis of these brains revealed that several genes critical for neuronal autophagy showed reduced expression levels in the BA22 region. Among the downregulated genes were ATG3, ULK2, and PI3KR4, which all play important roles in various aspects of macroautophagy [192]. A study by Merenlender-Wagner et al. proposed that neuronal dysregulation in schizophrenia could be linked to impairments in autophagy. The researchers demonstrated a significant reduction in mRNA levels of Beclin 1 (BECN1), a key autophagy-related protein, in the hippocampi of patients with schizophrenia. Additionally, the expression of BCL2, a protein that interacts with BECN1, was found to be altered in the same postmortem hippocampal tissue. Given that BECN1 is essential for initiating autophagy, its decreased expression may hinder the autophagic process in hippocampal neurons, limiting their ability to degrade damaged cellular components and contributing to neuronal dysfunction in schizophrenia [193].

Emerging evidence suggests that the dysregulation of autophagy could be a key mechanism via which abnormal circadian rhythms contribute to the onset of mania [194]. It is well established that autophagy is subject to rhythmic regulation, with circadian rhythms influencing autophagy at multiple stages, namely the activation of autophagy, the maturation of the phagophore, and the formation of the autophagosome, regulating autophagy in different ways. This supports the hypothesis that autophagy may play a crucial role in mediating the effects of circadian rhythm disturbances on mania-like symptoms [195,196,197]. When cells were treated with carbamazepine, a similar effect was observed in cells treated with the positive control rapamycin, indicating a significant increase in the number of autophagosomes and autophagic lysosomes in the treated cells, suggesting that carbamazepine enhances autophagy [198].

Medications used to treat various psychiatric disorders may exert their effects, at least in part, by inducing autophagy. For example, previous research has shown that lithium, a mood-stabilizing drug used for treating bipolar disorder, promotes autophagy [199]. This discovery led to further investigations into whether antipsychotic medications also influence autophagic activity in the brain. In a study by Zhang [200] a small-molecule screen revealed that three antipsychotics (fluspirilene, trifluoperazine, and pimozide) stimulate autophagy. This suggests that the downregulation of autophagic genes in specific brain regions, such as the BA22 region in patients with psychosis, might be at least partially reversed by antipsychotic treatments, which could enhance autophagic activity and boost the expression of autophagy-related proteins in these areas.

However, there is also evidence suggesting that autophagy induction may contribute to depressive-like behavior and cognitive impairments resulting from prenatal stress [201]. More recently, studies have shown that inhibiting autophagy can reduce the onset of depressive-like behavior induced by ecstasy in rats [202]. One of the earliest indications that antidepressants might influence autophagy was the observation of autophagy-related structures in the cytoplasm after treating cells with clomipramine [203]. This phenomenon could result from either the induction of autophagy or the inhibition of autophagic flux, which would impair functional autophagy. It is important to note that conclusions about active autophagy are often drawn based solely on the presence of autophagic markers, but this approach can be misleading without experiments that evaluate autophagic flux or the turnover of long-lived proteins [204]. More rigorous experiments later showed that desmethylclomipramine disrupts autophagic flux, thus inhibiting functional autophagy [205]. In contrast, amitriptyline was found to enhance autophagy in primary neurons and astrocytes, similar to the effect of citalopram, but venlafaxine did not appear to affect autophagy [206,207]. In a centralized presentation, the effect of psychiatric medication on autophagy is presented in Table 4.

### 4.5. Amyloid-Beta Peptides

Amyloid-beta (Aβ) is a peptide that serves as a key feature of Alzheimer’s disease (AD). Extracellular plaques comprise amyloid-β (Aβ) peptides, which are 40- or 42-amino acid fragments derived from amyloid-β protein precursor (AβPP). AβPP can be cleaved via two pathways: the non-amyloidogenic pathway involves α- and γ-secretases, producing soluble fragments, while in the amyloidogenic pathway, β-secretase first cleaves AβPP, followed by γ-secretase, releasing the Aβ fragment associated with Alzheimer’s disease [208]. The accumulation of Aβ peptides in the brain represents a neuropathological alteration that may take place up to 30 years prior to the appearance of obvious clinical symptoms and the formal diagnosis of AD [209,210].

Over the past two centuries, human diets have shifted from organic to industrialized foods, contributing to physiological changes and chronic diseases [211]. The Western diet (WD), high in fats and processed foods and low in fiber, has been linked to poor Aβ status in the brain, including low Aβ42 levels and increased amyloid accumulation [210,212]. This is largely due to metabolic syndrome and gut microbiota alterations, which affect the blood–brain barrier, neuroinflammation, and Aβ accumulation. In contrast, Mediterranean and ketogenic diets are associated with healthier Aβ levels and brain function [213,214].

Aβ accumulation in Alzheimer’s disease (AD) is linked to impaired brain energy metabolism, including mitochondrial dysfunction and reduced glucose metabolism. The ketogenic diet (KD), which promotes the use of ketone bodies as an energy source, has shown potential in addressing this issue [215,216]. However, while KD can lead to increased Aβ42 levels and may influence neuroinflammation, glucose metabolism, and mitochondrial function, it requires strict monitoring and may cause side effects. Ketone body supplementation is considered a suitable long-term alternative [217,218]. Additionally, KD is not recommended for individuals with liver or kidney conditions [219].

The link between alcohol consumption and Aβ levels is complex and varies by intake amount. Moderate alcohol consumption is associated with lower Aβ deposition, while higher intake increases Aβ accumulation, which may contribute to Alzheimer’s disease progression [210,220]. Research on alcohol’s effect on Aβ in humans is inconsistent, though animal studies show that high alcohol intake boosts Aβ production, while low-to-moderate intake may slow it down [221,222]. The impact of alcohol may also be influenced by its effect on inflammation, with moderate consumption reducing neuroinflammation and high consumption increasing it [223,224].

Carbohydrate consumption does not consistently have a direct link with Aβ status, but the type of carbohydrate matters [225]. High glycemic index carbohydrates, including fructose and sucrose, may increase insulin resistance, leading to neuroinflammation and Aβ accumulation [226,227]. On the other hand, low glycemic index carbohydrates are associated with better Aβ status [228]. Regarding lipids, the brain, which is rich in fats, is affected by serum cholesterol levels. High cholesterol, LDL, and triglyceride levels are linked to higher Aβ42 levels, while higher HDL levels correlate with lower Aβ42 levels [229].

Several studies have explored the relationship between vitamins and Aβ levels in Alzheimer’s disease (AD). Vitamin D has shown a positive correlation with Aβ42 in cerebrospinal fluid (CSF), and supplementation in AD patients reduced plasma Aβ42 levels [230]. In contrast, a study in older individuals without dementia found no association between vitamin D and Aβ [231]. For B vitamins, low B12 levels correlate with higher Aβ42, while folic acid supplementation may reduce Aβ42 levels [232,233]. Antioxidants such as Vitamin E, Vitamin C, and α-lipoic acid showed no significant changes in Aβ42 after supplementation in AD patients in [234]. In Table 5, we summarize the potential influence of vitamins on Aβ levels in Alzheimer’s disease.

## 5. Genetic Predisposition and Environmental Factors

### 5.1. Heritability of Psychiatric Disorders and Their Genetic Overlap

Estimates of heritability (the genetic contribution to trait variation) vary, with major depression showing 35% and schizophrenia showing over 60% [235]. The remaining variance is likely due to non-genetic factors, possibly including unidentified environmental influences. Another approach for assessing genetic and environmental contributions is using pedigree data (e.g., from parents, children, siblings) gathered from large national databases [236]. Furthermore, recent progress in research techniques has made it possible to leverage summary data from genome-wide association studies (GWASs) to estimate heritability. This is carried out by analyzing the linkage disequilibrium scores (LDSCs) of single-nucleotide polymorphisms (SNPs), which indicate how well a particular SNP is linked to other genetic variants [237].

Larger GWAS sample sizes have uncovered significant shared genetic risk factors across different psychiatric disorders, reflecting their overlapping clinical features [238]. A comprehensive meta-analysis of eight psychiatric disorders revealed 109 unique genetic loci linked to multiple conditions, with 11 of these loci exhibiting “discordant” effects, where they raised the risk of one disorder but lowered the risk of another [239].

A recent study highlighted a significant genetic overlap between intelligence (INT) and major depression (MD), identified using a condFDR/conjFDR approach. It discovered 92 genetic loci shared by both conditions, with half showing concordant allelic effects and half showing discordant effects, despite a low genetic correlation. The study also revealed that 88.3% of the genetic variants shared between INT and MD are causal, suggesting a strong polygenic relationship. In comparison, the overlap with height was much lower, indicating that the overlap between MD and INT is not primarily driven by omnigenic effects [240].

Gene-set analysis of the shared loci indicated that concordant effects were linked to cell adhesion and metabolism pathways, while loci with opposite effects were associated with gene silencing and neuronal development. These findings could provide insights for future neurobiological research [241]. Despite the lack of significant genetic correlation between INT and MD, the study showed a balanced distribution of loci with both concordant and discordant effects, suggesting a complex polygenic overlap between the two conditions [242,243].

Past studies’ results also pointed out that this overlap between INT and MD is similar to that seen between INT and bipolar disorder, contrasting with other psychiatric disorders such as schizophrenia, where most shared variants have detrimental effects on cognitive performance [244,245]. One study emphasized that genetic risk factors for MD influence cognitive traits, even in individuals not currently experiencing depression, underscoring the deep connection between cognitive functioning and psychiatric disorders [246]. These findings could inform theories of depression, especially how cognitive processes relate to the disorder’s negative thought patterns [247].

A positive genetic correlation between MDD and caudate volume suggests that genetic factors may influence both the caudate structure and the likelihood of treatment non-remission. This means that individuals with certain genetic predispositions, which affect caudate volume, may also be more likely to experience persistent depression that does not respond well to treatment. The caudate, involved in emotional regulation and the brain’s reward system, could, therefore, play a key role in determining how genetic factors impact treatment outcomes in MDD [248].

Recent studies have identified significant genetic variants related to clinical measures of autism spectrum disorder [249], ADHD [250], Tourette’s syndrome [251], anorexia nervosa [252], and depressive symptoms [253], as well as traits such as repetitive, restrictive behaviors [254] and social communication problems [255]. Replication studies were successful for ADHD and ASD, with the ASD GWAS identifying three loci and the ADHD GWAS identifying 12 loci. The direction of effect for these loci was confirmed in multiple independent cohorts. Functional annotation analyses of these GWAS results pointed to dopamine regulation and brain development as key factors in the etiology of ADHD and ASD. Notably, these findings were based on both adult and childhood samples, aiming to increase the power of detection [249,250].

PTSD has both environmental and genetic origins. While this is well established in behavioral genetics, molecular genetic data provide strong support for heritability, aligning with twin study results. Sex differences may act as environmental factors influencing how genetic variation is expressed, with biological factors such as sex hormones affecting PTSD susceptibility. If the female biological environment supports the expression of genetic variation while the male environment suppresses it, this could explain higher heritability estimates for PTSD in females. Research shows sex-based differences in trauma responses and environmental influences, suggesting that biological processes may cause a stronger genetic influence on PTSD in females than in males [256].

The study of genetic factors in bipolar disorder (BD) began with twin, family, and adoption studies, which revealed important insights about how family history influences the onset of mood disorders. Researchers found that the risk of developing BD is closely tied to a person’s genetic relationship with someone who already has the disorder. The closer the genetic link, the higher the risk, with the risk decreasing as the genetic distance from the affected individual increases [257].

One of the most telling studies came from Sweden, where researchers found that the risk of BD was significantly higher in relatives of those with the disorder. First-degree relatives had a risk 7.9 times greater than the general population, second-degree relatives had a risk 3.3 times greater, and third-degree relatives had a risk 1.6 times greater. This reinforced the idea that BD runs strongly in families [258].

Furthermore, these studies have shown that a family history of BD is often linked to an increased risk of experiencing other psychiatric disorders. Conditions such as schizophrenia, autism spectrum disorder (ASD), depression, anxiety, attention-deficit hyperactivity disorder, drug abuse, and personality disorders are more common in families where BD is present. Among these, the first three have the strongest genetic correlations with BD, highlighting a complex web of shared genetic risk across different mental health conditions [257,258,259].

Anxiety does not often stand alone. It frequently pairs with other psychiatric conditions, creating a tangled web of symptoms that complicates diagnosis and treatment. People with anxiety disorders often also struggle with depression, bipolar disorder, schizophrenia, or ADHD [260,261,262]. In fact, anxiety symptoms are so common in these disorders that they frequently exacerbate existing symptoms, leading to greater distress, poorer outcomes, and diminished quality of life for those affected [263,264].

The origins of anxiety disorders are multifaceted. Both genetics and environmental factors play significant roles in their development [265]. Researchers have found that genetic susceptibility is a major contributor, with twin studies estimating heritability to range from 30 to 50% [10]. Genome-wide association studies (GWASs) have revealed several genetic loci linked to anxiety disorders, highlighting the biological underpinnings of these conditions [266]. There is a compelling body of evidence suggesting that anxiety disorders share genetic liability with other psychiatric conditions [267]. However, while genetic correlation analyses have illuminated this overlap, they are not enough to capture the full genetic landscape [268].

Traditional methods do not always provide a comprehensive view of the genetic architecture shared between anxiety disorders and other psychiatric conditions. To bridge this gap, researchers are turning to innovative techniques such as bivariate causal mixture model (MiXeR), local analysis of covariant association (LAVA), and conditional false discovery rate (condFDR) methods. These advanced tools allow scientists to dig deeper into the genetic overlap, pinpointing specific risk loci and unlocking more precise insights into the biological pathways driving anxiety and its connections with other psychiatric disorders. Through these cutting-edge approaches, a clearer understanding of the genetic foundations of anxiety disorders is emerging, promising better, more targeted treatments for those affected [268,269,270,271].

A higher genetic risk of schizophrenia has been linked to poorer antidepressant responses, particularly in those with treatment-resistant depression (TRD). A study suggests that individuals resistant to antidepressants may carry a higher genetic burden for schizophrenia. Additionally, some evidence points to a genetic predisposition for major depression being associated with a poorer antidepressant response, though further validation is needed [272,273].

Genetic predisposition to autism spectrum disorder (ASD) has been linked to a less favorable response to cognitive behavioral therapy (CBT). This suggests that individuals with a higher genetic liability for ASD may face challenges in benefiting from this therapeutic approach, possibly due to unique traits associated with the disorder [274]. The same study also found that genetic predisposition for higher educational attainment is linked to better antidepressant response, likely reflecting socioeconomic factors. These findings suggest that both genetic and socioeconomic factors influence antidepressant efficacy, and future research could help to refine personalized treatment approaches [275].

### 5.2. Fine Mapping Variants in GWAS Loci

The year 2025 marks the 18th anniversary of psychiatric genome-wide association studies (GWASs), with the first psychiatric GWAS conducted in 2007 on bipolar disorder, funded by the WTCCC [276]. The fine mapping of variants in GWAS loci demands a thorough understanding of how specific variants influence a trait. It is essential to overcome linkage disequilibrium (LD) and pinpoint variants that are context-dependent and causally linked to the trait. This is crucial for unraveling disease mechanisms and accurately identifying the downstream genes and pathways impacted. A range of functional and computational (high-throughput) fine-mapping techniques have been developed and utilized to achieve this goal [277].

The cost of genotyping has significantly decreased, making GWAS an essential tool in uncovering genetic factors behind psychiatric disorders and traits. GWAS findings are often misinterpreted by the media, the public, clinicians, and researchers, necessitating a more rigorous and comprehensive review of the data to clarify these results [278,279].

Genetic pleiotropy refers to a single genetic variant affecting multiple traits [280]. Psychiatric conditions exhibit genetic pleiotropy, where common variants show moderate-to-high genetic correlations (rG) with schizophrenia, bipolar disorder, and major depressive disorder, with lower correlations with autism spectrum disorder (ASD) [249,281,282]. There are also moderate-to-mild genetic correlations between psychiatric conditions and traits such as neuroticism and fluid intelligence. Comparable correlations are observed at the brain transcriptomic level for these conditions [283,284]. Pleiotropy is not limited to common variants, as rare variants such as copy number variants (CNVs) are also associated with multiple psychiatric disorders and cognitive traits. Genotype–phenotype associations in children with copy number variants are associated with high neuropsychiatric risk in the UK [285]. Genetic and transcriptomic overlaps may extend to large-scale brain networks. Resting-state functional MRI (rs-fMRI) has revealed shared functional connectivity (FC) patterns among psychiatric disorders, particularly in the default mode, salience, and frontoparietal networks [286,287]. FC profiles of brain regions in individuals with rare CNVs (e.g., 16p11.2 and 22q11.2 deletions) overlap with those seen in psychiatric conditions such as ASD and schizophrenia, suggesting shared neural connectivity features. There is limited knowledge on how rare high-risk variants and polygenic scores (PGSs) for psychiatric conditions affect brain functional connectivity [288].

### 5.3. Polygenic Risk Scores

There is currently a lack of accurate models to predict the course of psychiatric disorders, making the ability to predict disorder progression highly valuable. Polygenic risk scores (PRSs) have shown potential for predicting the progression from at-risk states to clinical diagnoses in psychotic disorders. For example, schizophrenia PRSs are higher in individuals with first-episode psychosis later diagnosed with schizophrenia compared to those diagnosed with other psychotic disorders [289]. PRSs for multiple disorders (e.g., OCD, schizophrenia, MDD, bipolar disorder) have been found to predict subclinical symptoms, suggesting that they could help to identify individuals at higher risk of developing these conditions [290]. For disorders influenced by environmental factors, such as post-trauma psychopathology, PRSs could be used to screen at-risk groups (e.g., military personnel) for preventative measures. While PRSs currently have low accuracy and sensitivity for predicting individual risks, integrating them with other clinical data (e.g., psychiatric signs and symptoms) could improve prognostic accuracy in the future [291].

One study identifies specific SNPs, particularly those within calcium-channel activity genes, that are significantly associated with varied childhood- and adult-onset psychiatric disorders (schizophrenia, major depressive disorder, bipolar disorder, ADHD, autism spectrum disorder). Polygenic risk scores reveal cross-disorder associations, especially between adult-onset conditions, suggesting shared genetic risk across disorders. These findings underscore the pleiotropic effects of calcium channel signaling genes in psychopathology and highlight the potential of polygenic risk scores for improving our understanding of genetic underpinnings in psychiatry [292].

The genetic factors related to schizophrenia (SCZ) influence the response to lithium treatment in patients with bipolar affective disorder (BPAD). By analyzing genetic data from 2586 BPAD patients, researchers found that a higher genetic risk of SCZ (measured using a polygenic score) was linked to poorer lithium treatment response. Specifically, patients with a lower SCZ polygenic score responded better to lithium. Additionally, the same study identified genetic loci that may overlap in their effects on both SCZ susceptibility and lithium treatment response. These findings suggest that genetic factors could help to predict who will benefit from lithium treatment, supporting personalized treatment strategies in the future [293]. Pleiotropic variants, which affect multiple traits or conditions, can help to explain why certain side effects occur. For example, clozapine, an antipsychotic drug, can cause agranulocytosis, a dangerous drop in white blood cells. Interestingly, the same genetic variants linked to this side effect may also be associated with adverse reactions to statins, drugs commonly used to lower cholesterol. This connection highlights how a single genetic variant can influence the body’s responses to different medications, leading to seemingly unrelated side effects [294].

A PRS-TRS model developed using GWAS data from the CLOZUK and PGC datasets revealed that genetic factors contributing to treatment-resistant schizophrenia (TRS) are distinct from those associated with general schizophrenia liability. The PRS-TRS was linked to a history of clozapine use, indicating that individuals with a higher genetic risk of TRS are more likely to require this medication. Additionally, PRS-TRS showed correlations with cognitive traits, suggesting that the genetic factors underlying TRS are also associated with cognitive impairments in schizophrenia. This supports the idea that TRS has a specific polygenic basis separate from general schizophrenia risk [295].

A systematic review examined the relationship between polygenic risk scores (PRSs) and antidepressant response but found inconsistent results across multiple studies and traits. Furthermore, no significant association was found between PRS for MDD or schizophrenia and antidepressant response, even after analyzing data from several antidepressant trials [296]. This suggests that genetic risk scores may not reliably predict treatment outcomes for MDD [297].

One study found a significant association between PRS for ADHD and treatment-resistant depression (TRD), suggesting that undiagnosed ADHD could contribute to treatment resistance [298]. However, a subsequent study on patients treated with esketamine did not replicate these findings, showing no significant link between ADHD genetic risk and treatment response. This suggests that the impact of ADHD on treatment outcomes may depend on the specific antidepressant used [299].

Studies on PRS and ECT response found no significant link with PRS-MDD [300,301]. However, higher PRS-SCZ was associated with better outcomes, including greater symptom improvement and remission, aligning with evidence that psychotic features predict a positive response to ECT. Interestingly, this association differs from the lack of link between PRS-SCZ and pharmacological treatment outcomes, suggesting that genetic factors may influence responses to different types of therapies [301].

PRS for major depressive disorder (PRS-MDD) has been linked to poorer lithium response in bipolar disorder, with this association being most prominent in European and multi-ethnic patient samples. This suggests that genetic factors may influence how individuals with bipolar disorder respond to lithium therapy. However, the absence of a similar relationship in Asian populations highlights the genetic and ethnic variability in treatment responses, implying that genetic markers alone may not serve as universal predictors of lithium efficacy. These findings emphasize the need to explore genetic mechanisms further and understand how they interact with environmental and clinical factors to improve personalized treatment strategies [302].

A combined polygenic risk score (MET-PRS), integrating genetic data from schizophrenia, MDD, and BD, was developed to predict lithium response in bipolar disorder. The relevant study found that higher PRSs for schizophrenia (PRS-SCZ) and MDD were linked to poorer lithium response, while PRS for BD showed no such association. The refined model (MET2-PRS) significantly improved prediction accuracy. Patients in the highest PRS decile were 2.5 times more likely to be poor responders to lithium compared to those in the lowest decile. This highlights the value of combining multiple genetic factors to better predict treatment outcomes [303].

PRS for MDD explains only a small fraction of the variance in depression risk. For instance, a systematic review found that both MDD and bipolar disorder PRSs account for less than 2% of the variance in associated phenotypes, indicating their limited predictive capacity [304]. Moreover, most PRS studies have been conducted in populations of European descent, limiting their generalizability to other ethnic groups. This lack of diversity could lead to reduced accuracy and potential biases when applying PRSs across different populations. PRSs do not account for environmental factors, such as childhood trauma, that interact with genetic predispositions and can significantly influence the manifestation of genetic risks for depression, suggesting that PRSs alone may not fully capture an individual’s risk profile.

## 6. Neuromodulation

Neuromodulation refers to the process of altering or regulating nerve activity through the targeted delivery of electrical, chemical, or other stimuli to specific areas of the nervous system. It is used in both research and clinical settings to influence how the nervous system communicates and functions.

### 6.1. Non-Invasive Brain Stimulation

Non-invasive brain stimulation (NIBS) involves targeting specific brain regions using either direct electrical current or magnetic induction to create an electrical field on the scalp. This technique provides measurable outputs, such as motor evoked potentials, central transmission times, cortical silent periods, intracortical facilitation, and intracortical inhibition, utilizable for diagnostic purposes. Through repeated applications, NIBS modulates cortical excitability by either increasing or decreasing it. This repeated stimulation serves as a therapeutic intervention [305].

Non-invasive brain stimulation encompasses various techniques, with two primary examples being transcranial magnetic stimulation (TMS) and transcranial electrical stimulation (tES), with transcranial direct current stimulation (tDCS) being its most common variant. In TMS, a stimulation coil produces a rapidly alternating magnetic field (up to 3 T), which induces electric fields in both cortical and subcortical regions of the brain. In contrast, tES involves applying weak direct or alternating currents (in the milliamp range) through electrodes placed on the scalp [306].

Over the past two decades, notable advances have been made in understanding and refining NIBS targets. Focus has shifted from individual brain regions to targeting neural circuits, functional connectivity, and their dynamic states. NIBS interacts with the unique structural and functional characteristics of each brain, which differ across healthy and pathological states. While individual traits and disorder signatures may appear to be similar at a specific moment, they can potentially be distinguished over time through their progression and response to treatment [306].

#### 6.1.1. Transcranial Magnetic Stimulation

TMS operates through electromagnetic induction, where an electric current sent through a coil generates a magnetic field that induces a secondary electric field in conductive tissues, such as the brain. The rapidly changing magnetic field penetrates skin, bone, and fat with minimal resistance, allowing focused stimulation of brain regions. However, the intensity decreases with distance from the coil, limiting the depth of penetration [307].

The impact of a single TMS pulse on the motor cortex is evaluated using EMG-based motor evoked potentials (MEPs). While the pulse stimulates all neurons in the targeted region, their activation depends on factors such as the depolarization threshold, coil proximity, and orientation. Cortico-spinal pyramidal neurons produce high-amplitude direct waves (D waves), while interneurons produce smaller, delayed indirect waves (I waves). Variations in pulse intensity and coil positioning influence the patterns of D and I waves [308]. High-frequency rTMS (≥10 Hz) and intermittent theta-burst stimulation (iTBS) are typically considered excitatory, whereas low-frequency rTMS (≤1 Hz) and continuous theta-burst stimulation (cTBS) are viewed as inhibitory [308].

Repetitive TMS can occasionally trigger seizures under certain conditions. However, reported cases are rare (<0.1%), with no documented instances of irreversible complications or fatalities. The risk of seizures increases with higher resting motor thresholds (rMTs), greater stimulation frequencies, shorter intervals between trains, and longer train durations [309]. Low-frequency (LF) stimulation is recommended for patients with a history of seizures, as it is not linked to seizure induction and may even have protective effects [310]. Pain, discomfort at the application site, and headache are common adverse effects of rTMS [309,311]. Meta-analyses suggest that rTMS does not elevate the risk of treatment-induced mania or hypomania in patients with unipolar or bipolar depression [312,313].

Regarding major depressive disorder, the Canadian Network for Mood and Anxiety Treatments (CANMAT) recognizes rTMS as a first-line treatment for patients who have not responded to at least one antidepressant trial [314]. The suggested stimulation parameters are 110–120% of rMT (or 70–80% for TBS) delivered five times a week for 20–30 sessions, or fewer if a clinical response is seen. Low-frequency (LF), high-frequency (HF), and bilateral rTMS are all supported by strong evidence of their efficacy [315]. The effectiveness of rTMS and antidepressants appears to be comparable in patients with moderate-to-high levels of refractoriness. Two RCTs comparing rTMS with a full dose of venlafaxine found that both treatments showed equivalent efficacy [316,317]. Additionally, combining rTMS with antidepressant medications is more beneficial than rTMS alone [318]. Specific subtypes of MDD may have a preferential response stimulating either the left or right DLPFC. For example, patients with bipolar depression or depression accompanied by anxious symptoms may respond more favorably to LF-rTMS targeting the right DLPFC rather than HF-rTMS on the left DLPFC [319,320]. rTMS may improve not only depressive symptoms but also cognitive performance related to the pathophysiology of depression. A meta-analysis revealed that the cognitive benefits are modest and primarily affect visual scanning, psychomotor speed, and set-shifting abilities [321].

Regarding patients with schizophrenia, NIBS techniques have been applied to treat negative symptoms and auditory hallucinations (AH) [322,323]. For auditory hallucinations, a recent meta-analysis found a notable therapeutic impact of LF TMS over the left temporoparietal region; however, this has not been confirmed through large RCTs [324]. For negative symptoms, even though early findings were promising, including a meta-analysis suggesting efficacy, a recent RCT for 156 patients with schizophrenia and major negative symptoms found no superiority of HF-rTMS to the left DLPFC over 105 days of follow-up [323,325].

In the few studies conducted, it has been demonstrated that rTMS is also effective at treating generalized anxiety disorder, panic disorder, and PTSD [326,327,328].

#### 6.1.2. Transcranial Direct Current Stimulation

In tDCS, a low-intensity electric current (1–2 mA) is delivered through scalp electrodes, passing through multiple layers before reaching the gray matter. Due to high impedance in the skin, skull, and cerebrospinal fluid, only about 10% of the current reaches the brain, resulting in nonfocal stimulation [329]. Electrons move radially from the cathode to the anode. Early protocols developed by Nitsche et al. used 1 mA currents for 7–13 min, inducing excitatory (anodal) or inhibitory (cathodal) effects on motor cortex excitability, analyzing the results by measuring MEP amplitudes. While tDCS does not directly generate action potentials, it modulates synaptic transmission, influencing the frequency of endogenous neuronal firing [330].

Prior research suggests that the effects of tDCS on motor cortical excitability are not linear. Cathodal and anodal stimulation at 2 mA for 13 min produced excitatory effects, while that conducted at 1 mA for 26 min resulted in inhibitory effects [331,332].

The clinical effects of tDCS are influenced by several parameters, including electrode placement, sponge size, current intensity and polarity, stimulation duration, session count, and time between sessions [333,334]. Current intensity varies between 1 and 2 mA, with session durations typically ranging from 9 to 30 min [335].

Adverse effects are represented by paresthesia, tingling, skin redness, and discomfort at the application site, with erythema being the most common, occurring in over 80% of cases [335]. While patients often do not notice this effect, it may pose a challenge in double-blinded trials. No serious adverse events have been reported with tDCS [336].

The efficacy of tDCS in major depressive disorder was evaluated in a study called SELECT-TDCS, which examined transcranial direct current stimulation (tDCS) as an augmentative and substitutive treatment for antidepressants in 120 patients with moderate-to-severe depression [337]. Participants were divided into four groups: active tDCS + placebo, active tDCS + sertraline, sham tDCS + placebo, and sham tDCS + sertraline. Key findings included the following: (1) combined tDCS and sertraline treatment was the most effective; (2) active tDCS alone was superior to the placebo; and (3) tDCS was well tolerated, though five cases of hypomania/mania were noted in the combined group.

Another study, the ELECT-TDCS study, aimed to assess the noninferiority of tDCS compared to escitalopram (20 mg/day) in major depressive disorder, with a noninferiority margin set at 50% of escitalopram’s efficacy over placebo. The study lasted 10 weeks and included 22 tDCS sessions compared to the shorter SELECT-TDCS trial. A total of 245 patients were randomized to receive escitalopram, tDCS, or a placebo. ELECT-TDCS failed to demonstrate the noninferiority of tDCS to escitalopram. Superiority analyses showed that escitalopram was more effective than both tDCS and the placebo, while tDCS was superior to the placebo. The same study’s tDCS protocol involved an initial 3-week intensive phase followed by a taper, which may have influenced its outcomes. Comparisons with early rTMS studies suggest that longer stimulation protocols could improve efficacy [338].

Regarding the efficacy of tDCS in patients with schizophrenia, preliminary findings suggest that it may be beneficial in treating them. In a small RCT with 30 patients, anodal stimulation at T3P3 and cathodal stimulation at F3 led to reduced auditory hallucinations, with the effects persisting for three months [339]. Regarding treating negative symptoms of schizophrenia, small-scale studies suggest that tDCS, with anodal stimulation over the left DLPFC, may be effective, showing clinical improvement associated with increased connectivity between the left temporal gyrus and the left DLPFC [339,340].

tDCS has shown positive results in the treatment of addictions. Four small RCTs focused on nicotine (two studies), crack/cocaine, and alcohol dependence all reported positive outcomes [341,342,343].

NIBS techniques, particularly rTMS and tDCS, have seen increasing clinical use and significant progress in recent decades. The most promising results have been in treating MDD, with some efficacy also reported in schizophrenia. Their advantages include mild side effects and no absolute contraindications.

### 6.2. Electroconvulsive Therapy

ECT is a medical procedure that uses electrical currents to induce a seizure under general anesthesia for treating various psychiatric conditions such as severe depression, mania, catatonia, and clozapine-resistant schizophrenia. ECT also helps with suicidal ideation and can augment clozapine for psychosis. Strong evidence supports its use for treatment-resistant depression (TRD), and, overall, it is more effective than pharmacotherapy for depression and mood disorders, including bipolar depression and mania [344].

Electroconvulsive therapy (ECT) has undergone significant advancements since its introduction in the 1930s. Initially performed as unmodified ECT, it involved bilateral stimulation using a sine-wave voltage at power line frequency without anesthesia. Over time, the procedure has evolved into modified ECT, which is conducted under general anesthesia. Modern ECT typically employs right unilateral (RUL) stimulation with individually titrated ultra-brief or brief rectangular constant-current pulses, whereas bilateral brief pulse stimulation is typically used for treatment-resistant or urgent cases [344]. There are no absolute contraindications for using electroconvulsive therapy.

Concerning major depressive disorder, the response rate to ECT is 58% in patients who have experienced at least one antidepressant medication failure and 70% in those without. ECT is considered a first-line treatment for major depression in cases involving depression with psychotic features, such as delusions or hallucinations; persistent suicidal ideation with intent; severe weight loss, malnutrition, or dehydration due to refusal of food and fluids; catatonia; a history of positive response to ECT [345]; and pregnant women, particularly during the first trimester when antidepressant medication is considered contraindicated [346].

ECT has medical and cognitive side effects, though serious medical complications are rare. A review of patients with severe cardiovascular disease found that any complications were temporary and did not hinder treatment completion [347]. Commonly reported side effects include thirst or dry mouth, headache, nausea or vomiting, constipation, drowsiness, dizziness, muscle pain, temporary increases in blood pressure and cardiac arrhythmias [348], and dysuria in geriatric patients [349]. Cognitive impairments, particularly in autobiographical memory, can last up to three weeks post-ECT [348]. Studies show that greater symptom severity correlates with higher cognitive impairment, affecting attention, learning, memory, and executive function. While cognitive function typically returns to baseline after symptom remission, no further improvement is observed [350,351]. However, another study reported long-term memory improvements despite temporary post-ECT memory and verbal fluency issues [352]. Certain patient groups require careful evaluation before undergoing ECT, including elderly individuals with depression and those with comorbid borderline personality disorder [353].

Patients typically notice symptom improvement after approximately six ECT sessions [353]. A significant relapse rate occurs within the first six months, but this decreases notably with the continuation of pharmacotherapy or C-ECT compared to placebo. No significant differences in long-term relapse rates have been observed between unilateral and bilateral ECT. The highest success rate was found in patients who received pharmacotherapy continuation treatment following ECT [354].

ECT can be an effective augmenting strategy for treatment-resistant schizophrenia. A notable study by Petrides et al. investigated the effects of ECT augmentation in clozapine-resistant schizophrenia. In a randomized, single-blind trial, 39 patients with persistent psychotic symptoms despite clozapine treatment were assigned to either an ECT plus clozapine group or a clozapine-only group. ECT was administered bilaterally three times per week for four weeks, followed by twice-weekly sessions for another four weeks, while clozapine dosages remained unchanged. The same study defined response as a 40% or greater reduction in the Brief Psychiatric Rating Scale (BPRS) psychosis subscale, a Clinical Global Impression (CGI) severity rating of less than 3, and a CGI improvement rating below 2. The findings were striking—50% of patients in the ECT plus clozapine group met the response criteria, whereas none in the clozapine-only group showed improvement [355].

The risk of relapse following a successful acute course of electroconvulsive therapy (ECT) remains a significant clinical challenge, especially for patients with a history of treatment resistance. Retrospective studies suggest that continuation and maintenance ECT (C-ECT) can effectively reduce relapse risk and lower hospital readmission rates [353,354]. In Chanpattana et al.’s study, 58 patients who achieved remission during the acute phase were enrolled in a single-blind, six-month continuation treatment study. Patients were randomized into three groups: C-ECT with flupenthixol, flupenthixol alone, and C-ECT alone. After six months, relapse rates were significantly lower in the combination group (40%) compared to the monotherapy groups (93% each). These findings suggest that combining maintenance ECT with an antipsychotic may be effective for reducing relapse in treatment-resistant schizophrenia [355].

Despite its proven efficacy, ECT continues to face stigma and concerns regarding cognitive side effects. Future research should focus on optimizing treatment protocols to enhance efficacy while minimizing adverse effects. Continued investigation into individualized ECT strategies, including augmentation and maintenance therapies, will further refine its role in modern psychiatric care.

## 7. Neuroimaging

Unlike other medical fields, psychiatry still relies on clinical syndromes rather than biological markers for diagnosis. Brain imaging and genetic tests are used mainly to rule out other conditions, not to confirm psychiatric disorders such as depression or schizophrenia. However, the success of psychopharmacology suggests an underlying neurobiological basis. Rather than structural abnormalities, psychiatric illnesses likely stem from disrupted neural connectivity. While conventional imaging has been of little diagnostic use, advances in neuroimaging offer new insights into the brain’s role in mental illness [356].

Positron emission tomography (PET) has become an important tool in psychiatry, offering valuable insights into the neurochemical and metabolic processes underlying various psychiatric disorders and providing a more nuanced understanding of brain function beyond structural imaging [357].

Metabolic disturbances in schizophrenia may arise from disrupted connectivity within brain circuits, such as the thalamo-cortico-striatal system, leading to impaired metabolic coupling between regions [358]. Early studies showed a lack of normal metabolic coupling in patients with schizophrenia when stimulated, and more recent analyses revealed weaker correlations between the frontal lobe and other brain regions in medication-free patients [359]. FDG uptake in the frontal cortex and thalamus was negatively correlated with symptom severity, with lower metabolic asymmetry observed in patients compared to controls [360]. Additionally, negative symptoms were associated with lower FDG uptake, particularly in the right hemisphere, suggesting potential links with cerebral asymmetry in schizophrenia [361].

A less well-known finding is the two-fold increase in fluorodopamine turnover observed in patients with schizophrenia, detected through extended compartmental modeling applied to dynamic FDOPA recordings. This result suggests that dopamine is rapidly broken down despite its overproduction, leading to impaired signaling [362]. A similar effect is seen in healthy individuals treated with low doses of antipsychotics, which disinhibit dopamine synthesis [363]. However, in patients with schizophrenia on chronic antipsychotic treatment, reduced FDOPA trapping was observed, indicating a “depolarization block” effect [364,365]. This outcome has been supported by studies showing reduced FDOPA uptake in patients on stable antipsychotic medication, although a recent study on first-episode patients did not replicate these findings, possibly due to differences in illness phase or other factors [366].

An FDOPA study of treatment-resistant patients with schizophrenia and responders found elevated dopamine synthesis capacity in responders, suggesting that high dopamine synthesis is linked to a positive response to antipsychotics [367]. However, non-responders to second-line antipsychotics such as clozapine showed lower FDOPA uptake, which may indicate that reduced dopamine synthesis capacity is associated with poorer treatment response [368]. Longitudinal studies could clarify whether low FDOPA uptake is a trait or a response to medication. Additionally, machine learning applied to FDOPA PET scans in unmedicated patients has shown promise in predicting responses to first-line treatment [369].

A study used FDG-PET to explore brain metabolism in BD patients with and without a history of psychotic symptoms. The results revealed common FDG uptake deficits in areas such as the insula and temporal gyrus in BD patients compared to healthy controls. Notably, patients with a history of psychosis showed unique alterations in the right fusiform gyrus, suggesting that it could be a potential biomarker for psychosis in BD [370].

Early PET studies in depression using 15O-water revealed reduced blood flow in the anterior cingulate gyrus and left dorsolateral prefrontal cortex [371]. Another study with 15O-water showed decreased blood flow in the left medial prefrontal cortex and increased flow in the cerebellar vermis in depressed patients with cognitive impairment [372]. [18F]FDG PET scans demonstrated lower glucose metabolism in cortical, subcortical, and cerebellar regions in depressed individuals, including those experiencing a bipolar depressive episode [373,374]. Additionally, [11C]ABP688 PET revealed reduced mGluR5 binding in several brain areas, such as the prefrontal cortex and hippocampus, with decreased binding linked to more severe depression [375]. Dopamine dysfunction was also implicated in depression, as a study using MRI and [18F]FDOPA PET found lower dopamine uptake in the left caudate of patients with psychomotor retardation, suggesting dopamine’s role in the occurrence of these depressive symptoms [376].

PET imaging has also been utilized to monitor treatment responses in depression, revealing heightened 5-HT2A receptor activity in the frontal cortex as patients on SSRIs show improvement [377,378]. For those with treatment-resistant depression, PET scans showed that the combination of olanzapine and fluoxetine induced brain changes similar to those observed in patients who responded well to treatment [379]. A study examining ketamine’s effect on 5-HT1B receptors found that it alleviated depression symptoms in a manner inversely related to 5-HT1B receptor binding in the ventral striatum, suggesting that these receptors may be useful biomarkers for tracking ketamine treatment in depression [380].

There is currently widespread global enthusiasm in the field for using functional connectivity (FC) as a tool to discover clinical biomarkers, categorize patients into biologically distinct subgroups, identify potential treatment targets, monitor disease progression, and even predict future disease onset, advancement, and treatment responses [381].

There is no conclusive evidence that functional connectivity (FC) surpasses other imaging techniques, such as MRI or PET, in predicting risks or treatment responses. However, it holds promise for improving the understanding of disorders and forecasting symptom development. For instance, FC’s relationship with structural connectivity has been useful in detecting functional rigidity in individuals at risk of schizophrenia and bipolar disorder [382]. Additionally, integrating FC with methods such as PET-MRI has begun to uncover how neuroinflammation and FC together can reflect clinical symptoms [383]. For Alzheimer’s disease (AD), FC has provided new perspectives on the disease’s spread, particularly concerning tau and amyloid pathologies [384].

FC also shows potential for forecasting symptom changes when combined with other assessments. For example, FC alongside white matter integrity has been used to predict treatment response in social anxiety disorder [385] and monitor the progression from mild cognitive impairment to AD [386]. Moreover, FC plays a role in linking genetics and behavior, as seen in psychiatric disorders associated with serotonin-related gene variations [387]. Genetic factors, such as APOE in AD, influence FC patterns, which could assist in determining disease stage [384].

Following initial findings of ventricular enlargement via pneumoencephalography, extensive research has focused on using structural magnetic resonance imaging (MRI) to study schizophrenia [388]. These studies, when analyzed together, consistently show enlarged ventricles and reduced cortical volume, particularly in the temporal lobe and structures such as the hippocampus and amygdala. Additionally, MR spectroscopy has revealed metabolic disruptions in glutamate processing, redox balance (NAD+/NADH ratio), and mitochondrial function in those with schizophrenia [389,390].

Significant reductions in caudate volume have been observed in individuals with depression when compared to healthy controls [391]. This decrease in volume is associated with a loss of gray matter and potential disruptions in dopaminergic pathways, believed to contribute to the emotional dysregulation and severity of symptoms commonly seen in major depressive disorder (MDD) [392,393].

Some studies suggest that no difference in caudate volume exists between treatment responders and non-responders, though sex-specific effects have been hinted at [394]. Fronto-striatal atrophy, including caudate volume reduction, is commonly seen in treatment-resistant depression, as those with both reduced caudate volume and high anhedonia tend to experience more persistent treatment non-response [395,396,397]. The caudate nucleus is central to the brain’s reward system, and dysfunction in this system, particularly in dopaminergic pathways, is thought to contribute to anhedonia in MDD [398,399]. Higher dopaminergic receptor availability in the striatum, including the caudate, has been found in MDD patients, especially non-remitters, suggesting dopaminergic dysfunction in treatment response [400].

Since its discovery 24 years ago, the default mode network (DMN) has become a key topic of focus in cognitive research, enhancing our understanding of brain organization, its role in cognition and emotion, and its potential link to psychopathology [401,402].

The brain’s default mode network, consisting of regions such as the posterior cingulate cortex (PCC), medial prefrontal cortex (MPFC), and inferior parietal lobule (IPL), is closely linked to self-referential mental activity. Initially identified through imaging studies showing higher activity during passive tasks, the DMN has been associated with a range of self-related processes, including self-referential thought and monitoring of the environment, body, and emotional state [403,404].

The DMN is linked to attention lapses that disrupt goal-directed behavior. Inadequate DMN suppression impairs selective attention and stimulus processing, with reduced deactivation in key DMN regions and hyperactivity in frontal and parietal areas, underscoring its role in cognitive tasks through interaction with other brain networks [405].

Individuals at risk of depression may be more likely to engage a self-referential brain network when processing negative information rather than positive information. This bias toward negative information is associated with ruminative thinking and could reflect an underlying neurocognitive vulnerability to depression. Targeting brain regions such as the MPFC or IPL could offer potential preventative interventions for those at risk of developing depression [406].

The DMN is essential for memory operations, particularly for personally relevant details. Early studies of Alzheimer’s patients showed reduced hippocampus–DMN coactivation, linking the network to memory processing [407,408]. Subsequent research consistently highlights the involvement of DMN nodes in memory encoding, free recall, and retrieval, especially in autobiographical memory [409].

In conclusion, research on patients with psychiatric disorders has highlighted the role of DMN in stimulus-independent thoughts. Disruptions in these thoughts have been linked to symptoms such as hallucinations and depression, underscoring the DMN’s significance for both normal cognitive function and the development of psychopathology [410,411].

## 8. Conclusions

Major depressive disorder (MDD) remains one of the most prevalent and disabling psychiatric disorders globally, with a multifactorial etiology involving complex interactions between genetic, biological, and environmental factors. Despite decades of research, the precise molecular mechanisms underpinning MDD are not fully understood due to the disorder’s clinical heterogeneity, variable age of onset, differing courses, and frequent comorbidities.

Advancements in genomic technologies, particularly large-scale genome-wide association studies (GWASs), have significantly contributed to our understanding of MDD’s genetic underpinnings. These studies have identified over 100 genetic loci associated with MDD, implicating biological pathways related to synaptic signaling, neuronal development, immune function, and the hypothalamic–pituitary–adrenal (HPA) axis. However, the effect sizes of individual variants remain small, and no single genetic marker is currently robust enough to serve as a clinical predictor.

In response, the use of polygenic risk scores (PRSs) has been developed to quantify the cumulative impact of many genetic variants. While PRS has shown potential as a research tool to assess genetic liability, its clinical utility is still limited. Current MDD PRSs explain only a small portion of disease heritability and perform modestly in predicting risk at an individual level. This limitation is due to several factors, namely small variant effect sizes, phenotypic variability in GWAS cohorts, and the under-representation of non-European populations, which affect generalizability.

Furthermore, PRS models currently neglect key environmental risk factors such as early life stress, trauma, and ongoing psychosocial adversity—factors that are well-documented contributors to MDD. The interaction between genes and environment (GxE), as well as epigenetic mechanisms such as DNA methylation and non-coding RNA regulation, are essential to understanding how genetic susceptibility is modulated by life experiences. Their absence from current risk models represents a critical gap in fully elucidating the pathophysiology of MDD.

Nevertheless, insights from genetic studies are increasingly contributing to the field of personalized psychiatry, especially for treatment. MDD is often difficult to treat, with a substantial portion of patients showing resistance to first-line antidepressants. In this context, non-pharmacological interventions such as repetitive transcranial magnetic stimulation (rTMS) have emerged as promising therapeutic alternatives, particularly for treatment-resistant depression (TRD). rTMS is a neuromodulatory technique that uses magnetic fields to stimulate specific regions of the brain, most commonly the left dorsolateral prefrontal cortex (DLPFC), which is associated with mood regulation. Clinical trials and meta-analyses have demonstrated its efficacy and safety, with response rates ranging between 30% and 60% in TRD populations. The appeal of rTMS lies in not only its non-invasive nature but also its favorable side-effect profile compared to pharmacotherapy.

Moreover, emerging research suggests that genetic markers may influence rTMS response, raising the possibility of genetically informed treatment selection. For example, polymorphisms in genes related to neuroplasticity, such as BDNF (brain-derived neurotrophic factor), as well as those influencing glutamatergic neurotransmission, have been associated with differential responses to rTMS. This line of inquiry holds promise for integrating PRS or individual gene variants into algorithms that predict treatment outcomes, potentially guiding clinicians in selecting rTMS or other neuromodulatory treatments for patients with specific genetic profiles.

Other innovative therapeutic approaches, including ketamine/esketamine infusions, vagus nerve stimulation (VNS), deep brain stimulation (DBS), and digital cognitive behavioral therapy (dCBT), are also being explored, often in tandem with genomic or neuroimaging biomarkers, to refine patient selection and improve efficacy.

In conclusion, while identifying genetic risk loci and developing PRS represent important milestones in our understanding of MDD, substantial limitations remain. These include low predictive accuracy, population-specific biases, limited consideration of environmental and epigenetic influences, and a gap between genetic findings and clinical application. However, as we move toward a more integrative model of psychiatry—one that combines genomic data with neurobiological, psychological, and environmental information—the role of genetics in guiding personalized, targeted interventions, such as rTMS, is likely to expand. Future research should prioritize longitudinal, multi-omic, and multi-ethnic studies that can enhance the accuracy of PRS and facilitate integrating genetic insights into individualized treatment strategies for MDD.

## Figures and Tables

**Table 1:** Correlations between neuroticism and generalized anxiety disorder in monozygotic and dizygotic twin pairs stratified by sex ^a^ Reproduced with permission from John M. (Jack) Hettema, MD, PhD, Professor, 2025 [15].

Twin Pair	Twin 1 Neuroticism	Twin 1 Generalized Anxiety Disorder	Twin 2 Neuroticism	Twin 2 Generalized Anxiety Disorder
Male–male				
Twin 1: neuroticism		0.318	0.358	0.206
Twin 1: generalized anxiety disorder	0.394		0.174	0.128
Twin 2: neuroticism	0.207	0.074		0.394
Twin 2: generalized anxiety disorder	0.064	0.093	0.316	
Female–female				
Twin 1: neuroticism		0.569	0.303	0.327
Twin 1: generalized anxiety disorder	0.195		0.214	0.150
Twin 2: neuroticism	0.119	0.142		0.462
Twin 2: generalized anxiety disorder	0.229	0.186	0.468	
Opposite-sex				
Twin 1: neuroticism				
Twin 1: generalized anxiety disorder	0.339			
Twin 2: neuroticism	0.113	0.095		
Twin 2: generalized anxiety disorder	0.038	0.059	0.211	

^a^ Monozygotic pairs are above each black diagonal; dizygotic pairs are below each black diagonal.

**Table 2:** Other genes thought to be associated with schizophrenia.

Gene Symbol	Gene Description	Chromosome	Functions
APOE	Apolipoprotein E	19	A main apoprotein of the chylomicron that binds to a specific receptor on liver cells and peripheral cells; essential for the normal catabolism of triglyceride-rich lipoprotein constituents
CFTR	Cystic fibrosis transmembrane conductance regulator (ATP-binding cassette subfamily C, member 7)	7	Involved in multidrug resistance; functions as a chloride channel and controls the regulation of other transport pathways
CNTNAP2	Contactin-associated protein-like 2	7	A member of the neurexin family; functions in the vertebrate nervous system as a cell adhesion molecule and receptor
COMT	Catechol-O-methyltransferase	22	Catecholamine neurotransmitter metabolism; VCFS region (22q deletion syndrome)
DISC1	Disrupted in schizophrenia 1	1	Neurite outgrowth and cortical development; disrupted in t(1;11)(q42.1;q14.3)
DRD2	Dopamine receptor D2	11	G protein-coupled receptor for dopamine; inhibits adenylyl cyclase
DTNBP1	Dystrobrevin-binding protein 1	6	A component of the biogenesis of lysosome-related organelles complex 1
ERBB4	V-erb-a erythroblastic leukemia viral oncogene	2	Receptor for neuregulins; cell differentiation
FMR1	Fragile X mental retardation 1	23	May be involved in nucleus → cytoplasm mRNA trafficking
HTR2A	5-hydroxytryptamine (serotonin) receptor 2A	13	G protein-coupled receptor for serotonin; activates phosphoinositide hydrolysis
NRG1	Neuregulin 1	8	Signaling protein that mediates cell–cell interactions; has roles in growth and development
NRGN	Neurogranin	11	Postsynaptic protein kinase substrate; learning and memory; glutamate signaling
NRXN1	Neurexin 1	2	Functions in the nervous system as a cell adhesion molecule and receptor
PARK7	Parkinson’s disease (autosomal recessive, early onset) 7	1	Positive regulator of androgen receptor-dependent transcription; apparently protects neurons against oxidative stress and cell death
SCNA	Synuclein, alpha (non-A4 component of amyloid precursor)	4	May serve to integrate presynaptic signaling and membrane trafficking
TCF4	Transcription factor 4	18	Neuronal transcriptional factor; neurogenesis
VIPR2	Vasoactive intestinal peptide receptor 2	7	Peptide that functions as a neurotransmitter and a neuroendocrine hormone
ZNF804A	Zinc finger protein 804A	2	Transcription factor; neuronal connectivity in the dorsolateral prefrontal cortex

**Table 3:** CYP enzyme activity and its effect on diazepam metabolism [92,93].

Genotype	Enzymatic Activity	Impact of Diazepam	Clinical Consequences
CYP2C19 PM (Poor Metabolizer)	Low/Absent	Slow metabolism, drug accumulation	Increased risk of adverse reactions (dependence, tolerance)
CYP2C19*2 (Allele)	Reduced activity	Slow metabolism, drug accumulation	Higher risk of side effects
CYP2C19*17 (Allele)	Increased activity	Rapid metabolism, faster drug clearance	Reduced efficacy, possible higher dose needed
CYP2B6 PM (Poor Metabolizer)	Low	Slow metabolism, drug accumulation	Possible need for dose reduction

**Table 4:** The effects of different psychiatric medications on autophagy.

Medication	Type	Effect on Autophagy	Possible Mechanism/Notes
Lithium	Mood stabilizer	Promotes autophagy	Enhances autophagic activity in the brain
Fluspirilene	Antipsychotic	Stimulates autophagy	Identified in a small-molecule screen
Trifluoperazine	Antipsychotic	Stimulates autophagy	May help reverse the downregulation of autophagic genes in the BA22 region
Pimozide	Antipsychotic	Stimulates autophagy	Could enhance the expression of autophagy-related proteins
Clomipramine	Antidepressant (TCA)	Presence of autophagy-related structures, but unclear effect	May induce or inhibit autophagic flux; further experiments are required
Desmethylclomipramine	Metabolite of clomipramine	Inhibits functional autophagy	Disrupts autophagic flux
Amitriptyline	Antidepressant (TCA)	Enhances autophagy	Observed in primary neurons and astrocytes
Citalopram	Antidepressant (SSRI)	Enhances autophagy	Similar effect to amitriptyline
Venlafaxine	Antidepressant (SNRI)	No significant effect on autophagy	Does not appear to alter autophagic processes

**Table 5:** Vitamins and their potential influence on Aβ levels in Alzheimer’s disease.

Vitamin	Effect on Aβ Levels	Notes
Vitamin D	↓ Plasma Aβ42 in AD patients after supplementation	Positive correlation with Aβ42 in CSF; no association in older adults without dementia
Vitamin B12	Low levels correlate with ↑ Aβ42	Deficiency may contribute to higher Aβ accumulation
Folic Acid	Supplementation may ↓ Aβ42 levels	Potential protective effect
Vitamin E	No significant change in Aβ42 after supplementation in AD patients	Antioxidant properties but no direct effect on Aβ levels observed
Vitamin C	No significant change in Aβ42 after supplementation in AD patients	Similar to vitamin E; no observed reduction in Aβ
α-Lipoic Acid	No significant change in Aβ42 after supplementation in AD patients	Antioxidant effects but no impact on Aβ levels

Legend: ↓ = decrease; ↑ = increase.
